# Human adaptation to high altitude: a review of convergence between genomic and proteomic signatures

**DOI:** 10.1186/s40246-022-00395-y

**Published:** 2022-07-15

**Authors:** Vandana Sharma, Rajeev Varshney, Niroj Kumar Sethy

**Affiliations:** Peptide and Proteomics Division, Defence Institute of Physiology and Allied Sciences (DIPAS), Defence Research and Development Organisation (DRDO), Lucknow Road, Timarpur, Delhi 110054 India

**Keywords:** High altitude, Hypoxia, High-altitude adaptation, Genomics, Proteomics

## Abstract

Both genomics- and proteomics-based investigations have identified several essential genes, proteins, and pathways that may facilitate human adaptive genotype/phenotype in a population-specific manner. This comprehensive review provides an up-to-date list of genes and proteins identified for human adaptive responses to high altitudes. Genomics studies for indigenous high-altitude populations like Tibetans, Andeans, Ethiopians, and Sherpas have identified 169 genes under positive natural selection. Similarly, global proteomics studies have identified 258 proteins (± 1.2-fold or more) for Tibetan, Sherpa, and Ladakhi highlanders. The primary biological processes identified for genetic signatures include hypoxia-inducible factor (HIF)-mediated oxygen sensing, angiogenesis, and erythropoiesis. In contrast, major biological processes identified for proteomics signatures include 14–3-3 mediated sirtuin signaling, integrin-linked kinase (ILK), phosphoinositide 3-kinase (PI3K)/protein kinase B (AKT), and integrin signaling. Comparing genetic and protein signatures, we identified 7 common genes/proteins (*HBB*/hemoglobin subunit beta*, TF*/serotransferrin*, ANGPTL4*/angiopoietin-related protein 4*, CDC42*/cell division control protein 42 homolog*, GC*/vitamin D-binding protein*, IGFBP1*/insulin-like growth factor-binding protein 1, and *IGFBP2*/insulin-like growth factor-binding protein 2) involved in crucial molecular functions like IGF-1 signaling, LXR/RXR activation, ferroptosis signaling, iron homeostasis signaling and regulation of cell cycle. Our combined multi-omics analysis identifies common molecular targets and pathways for human adaptation to high altitude. These observations further corroborate convergent positive selection of hypoxia-responsive molecular pathways in humans and advocate using multi-omics techniques for deciphering human adaptive responses to high altitude.

**Supplementary Information:**

The online version contains supplementary material available at 10.1186/s40246-022-00395-y.

## Introduction

The nonlinear decrease in barometric pressure with increasing altitude decreases the partial pressure of oxygen in the ambient air. As a result, humans ascending to high altitude (HA, defined as 2500 m and above sea level) inhale fewer oxygen molecules per breath. However, the oxygen percentage in the atmosphere remains static. This condition of hypobaric hypoxia is the principal driver of an array of physiological and pathological changes experienced by lowlanders engaged in professional, pilgrimage, and recreational activities at HA [16, 31, 99]. Other environmental changes associated with HA like cold, increased UV radiation exposure, extremely low absolute humidity, and limited food resources also affect health and quality of life. Despite this, high mountain regions have been inhabited over millennia and have witnessed an increased inflow of sojourns in recent years. According to a recent global population estimate, 500.3 million humans live at ≥ 1500 m, 81.6 million at ≥ 2500 m, and 14.4 million at ≥ 3500 m, and there are several areas of habitation at over 4000 m. Most of these populations reside in three central regions: the Himalayas of South-Central Asia, the Andes of South America, and the highlands of Eastern Africa [35, 72, 91, 100]. These high-altitude populations, namely Tibetans, Sherpas, Ladakhis, Andeans, and Ethiopians, have developed diverse physiological adaptive responses to counter the ill effects of persistent hypobaric hypoxia [8, 10, 11, 38, 101]. Notably, Tibetan highlanders exhibit elevated resting ventilation compared to Andean highlanders and low-altitude populations at low altitude. Tibetans express a normal hypoxic ventilatory response (HVR) whereas Andean highlanders have lower HVRs than lowlanders [8]. In addition, Tibetan highlanders have less pulmonary vasoconstrictor response to hypobaric hypoxia than Andeans and Ethiopians [71]. Andean high-altitude natives possess higher hemoglobin concentrations as compared to lowlanders at similar altitudes. In contrast, Tibetans have lower hemoglobin concentrations as compared to Andeans that correlates with reproductive success and exercise capacity. During pregnancy, Tibetans and Andeans demonstrate more significant pelvic blood flow redistribution favoring uteroplacental circulation and higher uterine artery volumetric flow, resulting in higher oxygen delivery than lowlanders [71]. Himalayan high-altitude native Sherpas have higher plasma volume than Andeans resulting in a comparable total blood volume at a lower hemoglobin concentration representing new adaptive phenotypes at high altitude [88].

Lowland residents (≤ 1500 m) visiting high altitudes elicit an integrated set of physiological (including respiratory and cardio-pulmonary) and hematological responses for successful acclimatization to high altitude [7, 73]. The ventilatory acclimatization of lowlanders is characterized by progressive increases in ventilation, arterial oxygen partial pressure, and oxygen saturation (SaO_2_), along with normalization of arterial pH. It is generally believed that increased ventilation and decreased total body water (resulting in a reduced plasma volume) are the two vital physiological components of high-altitude acclimatization. A concomitant decrease in total body water content and reduction in plasma volume results in hemoconcentration facilitating the oxygen-carrying capacity of the blood. The net result of the increased ventilation and hemoconcentration is a near normalization of arterial oxygen content after an approximately 7-day residence at a high altitude. It is generally accepted that increased ventilation and hemoconcentration facilitate near normalization of lowlander arterial oxygen content after an approximately 7-day residence at a high altitude [73, 82]. Failure to acclimatize results in several high-altitude maladies, namely acute mountain sickness (AMS), high-altitude cerebral edema (HACE), and high-altitude pulmonary edema (HAPE) [26, 97]. In recent years, the ever-increasing annual footfall of lowlanders at high altitude comprising of army personnel, government officials, miners, pilgrims, trekkers, and porters emphasizes an increased understanding of high-altitude acclimatization and adaption representing an essential branch of public health [5].

With the multi-omics approach in hypoxic research, many studies are being carried out to understand high-altitude genomic, proteomic, and metabolic perturbations, including ascending lowlanders and acclimatized lowlanders and indigenous high-altitude populations [55]. Using whole-genome sequencing, genome-wide association studies (GWAS), and genome-wide scans of DNA polymorphism, many studies have identified several loci under positive selection, mainly in Tibetans, Andeans, and Ethiopians, possibly conferring adaptive hypoxia phenotypes [18, 33, 45]. Independent research groups using different experimental approaches and methods have identified such loci in a population-specific manner, indicating repeated selection of genotypes that confer similar adaptive phenotypes under similar environmental hypoxia conditions. In recent years, proteomics-based studies for both acclimatized lowlanders and native high-altitude populations have complemented genomics endeavors, albeit the number of studies and sample size are on the lower side. It is important to note that most of the high altitude–proteomics endeavors have targeted at identifying proteins and molecular pathways underlying lowlander adaptive and maladaptive responses at high altitude. In addition, both genomics and proteomics studies have identified shared and unique biological pathways for indigenous highlanders despite of the same environmental stressors at high altitude.

This literature review summarizes the similarities and differences between genomics and proteomics studies to provide comprehensive information on human adaptation to high altitude. We provide an up-to-date appraisal of the literature for positively selected human genes irrespective of ancestry and human proteins identified from blood, plasma, serum, saliva, muscle tissue, and urine. Finally, we have evaluated convergence between genomics and proteomics studies by identifying common genes/proteins and associated molecular pathways providing an integrated molecular response facilitating acclimatization to high altitude.

## Methods

A sensitive search strategy was constructed to locate publications relevant to human response to high altitude. We used the keywords high-altitude, high-altitude genomics, gene selection signatures for high-altitude hypoxia adaptation, Tibetan genomics, Andean genomics, Ethiopian genomics, Kyrgyz genomics, high-altitude proteomics, high-altitude hypoxia acclimatization proteomics, high-altitude muscle proteomics, Tibetan proteomics, Andean proteomics, and Sherpa proteomics to search the following databases: Medline (OVID), EMBASE (OVID), PUBMED, Scopus and Google Scholar. The criteria for exclusion included abstracts, editorials, narrative reviews, case reports, letters to the editor, meta-analyses, and animal and cell culture studies. Two independent reviewers scrutinized the results and systematically presented them in the form of tables—high altitude–proteomics studies, including temporal studies for human volunteers exposed to high altitude. Proteins identified using only global proteomics studies reporting high-altitude acclimatization and adaptation were filtered out and included in the present study. High throughput proteomics studies with supplementary information for total proteins identified were included for IPA (Ingenuity Pathway Analysis)-based pathway mining. Proteins which reportedly showed an up or downregulation of the fold change cutoff (± 1.2-fold) were used for IPA-based pathway analysis. Similarly, studies for varying high altitudes with paired samples, identified proteins with *p* value ≥ 0.05 (paired t test) were included for IPA analysis. Further, the protein markers were categorized in study time scales for human blood and skeletal muscle studies. Top canonical pathways associated with each time scale for plasma and serum and skeletal muscle tissue were identified using IPA (Ingenuity Pathway Analysis, QIAGEN Inc., https://digitalinsights.qiagen.com/IPA). A comparison analysis was performed using available AMS (Acute Mountain Sickness) positive and AMS negative proteome profiles in the literature and the top 5 canonical pathways were identified using IPA software (Table 1). Genomics studies from Tibetan/Sherpa, Andeans, Ethiopian, and Kyrgyz high-altitude populations were screened. Genes showing signals of selection in high-altitude native populations were manually screened with a cutoff p value. Both the gene and protein lists were compared using the IPA compare analysis feature and Venny 2.1.0 (https://bioinfogp.cnb.csic.es/tools/venny/) to identify common signatures. IPA-based analysis was performed to identify top 15 canonical pathways from the common signatures (Additional file 1).

**Table 1:** A comparative presentation of pathways identified for AMS-R and AMS-S phenotypes using IPA

Sr. no	Ingenuity canonical pathways	−log *p* value	Molecules
*AMS-R*			
1	Complement System	14.9	C4A/C4B, C5, C6, C7, C8A, C8B, C8G, CFH
2	Acute-Phase Response Signaling	7.5	APCS, C4A/C4B, C5, CRP, ITIH3, KLKB1, RBP4
3	LXR/RXR Activation	7.11	APOA5, APOE, C4A/C4B, GC, HPR, RBP4
4	FXR/RXR Activation	5.56	APOE, C4A/C4B, GC, HPR, RBP4
5	Systemic Lupus Erythematosus Signaling	4.09	C5, C6, C7, C8A, C8B, C8G
*AMS-S*			
1	Complement System	9.34	C1QBP, C4A/C4B, C6, C8A, C8B, C8G, CFH
2	LXR/RXR Activation	9.34	APOA5, APOE, C4A/C4B, GC, HPR, KNG1, RBP4
3	EIF2 Signaling	4.96	EIF3B, RPL10A, RPL18A, RPS10, RPS14, RPS25, RPS26, RPS27
4	Regulation of elF4 and p70S6K Signaling	4.56	EIF3B, ITGB1, RPS10, RPS14, RPS25, RPS26, RPS27
5	FXR/RXR Activation	4.42	APOE, C4A/C4B, GC, HPR, KNG1, RBP4

The table represents the top 5 identified canonical pathways (with *p* values) and contributing proteins for both AMS-R and AMS-S phenotypes

## Results and discussion

### Proteomics for high-altitude acclimatization

HA acclimatization consists of integrated physiological, biochemical, and molecular changes in native lowlanders for quickly adapting to compromised oxygen availability. Individual acclimatization to hypobaric hypoxia depends on external or environmental factors (rate of ascent, mode of high-altitude induction, severity, and duration of hypoxic stress) and individual response (including cellular oxygen transport, intensity oxygen consumption, metabolic characteristics, and behavioral reactions). These combined factors determine whether the body successfully acclimatizes to HA or is overwhelmed by hypobaric hypoxia and develops pathologies. The following section will discuss lowlander response to acute exposure followed by chronic exposure to HA.

#### Plasma and serum proteomics

Studies reporting human exposure to short-term hypoxia exposure (30 min and 9 h).

have either used simulated chambers [34, 53] or real HA exposure (H. [66, 67] to study blood plasma and serum proteins after exposure. Evaluating the effect of acute hypoxia exposure of 30 min in a simulated altitude (2400 m or 8,000 ft) equivalent to cabin pressure during airline travel, Hinkelbein et al. studied the serum proteome of 10 volunteers (9 males and 1 female) using 2D DIGE (2- Dimensional Differential Gel Electrophoresis) followed by MALDI-TOF (Matrix Assisted Laser Desorption/Ionization- Time of Flight). The authors identified 10 proteins, out of which higher levels of 6 proteins, including apolipoprotein E (APOE), complement C1q subcomponent subunit precursor A (C1QA), complement C1q subcomponent subunit precursor B (C1QB), clusterin precursor (CLU), prothrombin (F2) and glyceraldehyde 3 phosphate dehydrogenase GAPDH) and lower level of 4 proteins comprising albumin (ALB), phosphoglycerate kinase 1 (PGK1), catalase (CAT) and carbonic anhydrase 1 (CA1) were observed as compared to pre-exposure levels.

Two independent studies exposed lowlanders to 9 h of hypobaric hypoxia and screened the volunteers based on LLQ (Lake Louise Questionnaire) scoring for AMS resistance. Using the discovery plasma proteomics approach, the authors identified proteins associated with AMS resistance and thus facilitating high-altitude acclimatization [53, 66, 67]. Julian et al. exposed 20 healthy volunteers (17 men and 3 women) living near Denver, Colorado (1609 m) for 9 h of hypobaric hypoxia (4875 m; 425 mmHg) in a hypobaric chamber [53]. Using pooled plasma samples of AMS-resistant volunteers with two different nano-liquid chromatography–tandem mass spectrometry platforms, the authors identified 350 proteins or protein fragments for both the studied groups. 19 proteins were identified (fold change ≥ 1.8) between pre-ascent and post-9 h hypoxia exposure. Out of these 19 proteins, 8 proteins, including serum amyloid P component (SAMP), insulin-like growth factor-binding protein complex acid-labile subunit (IGFALS), complement component 7 (C7), mannose-binding protein C (MBL2), apolipoprotein C4 (APOC4), serum amyloid A4 protein (SAA4), apolipoprotein C1 (APOC1) and von Willebrand factor (VWF) were unique to AMS-resistant group. Extending this 9-h hypoxia exposure protocol to real high-altitude exposure, Lu et al. exposed healthy lowlanders to 3800 m height and identified AMS-resistant and AMS-susceptible volunteers using Lake Louise Questionnaire Scoring [66, 67]. Comparing the post-ascent plasma proteome with the pre-ascent proteome, the authors identified 89 proteins (fold change > 1.5) for AMS-susceptible patients, out of which 17 proteins were upregulated and 72 were downregulated. Similarly, for AMS-resistant volunteers, the authors identified 421 proteins, out of which 61 were upregulated and 360 downregulated proteins. Interestingly, AMS-resistant volunteers exhibit lower plasma levels of 10 key TCA cycle metabolic enzymes, namely, pyruvate dehydrogenase alpha 1 (PDHA1), dihydrolipoyl dehydrogenase (DLD), ATP-citrate synthase (ACLY), aconitase (ACON), isocitrate dehydrogenase (IDH1, IDH2, IDH3A, IDH3B), 2-oxoglutarate dehydrogenase (OGDH), dihydrolipoyl lysine-residue succinyltransferase (DLST), succinyl-CoA ligase (SUCLG1, SUCLG2, and SUCLA2), succinate dehydrogenase (SDHA, SDHB), and malate dehydrogenase (MDH2). 11qIn addition, lower plasma levels of proteins belonging to the ribosome (Rps2p, Rps9p, Rps24p, Rpl15p) and proteasome (PSMC3, PSMA3, PSMD12, PSMD1, PSMD2, PSMD11, PSMA7, PSMC4, PSMB1, PSMA2, PSMA1, PSMC5, PSMA6, PSMD7) pathway were also identified (Table 1).

We have picked out several molecular pathways for AMS-resistant and -positive volunteers (Table 1). We recognized that EIF2 signaling, regulation of EIF4 and p70SCK signaling, mTOR signaling, sirtuin signaling pathway, TCA cycle, and gluconeogenesis are predicted to be activated in AMS-positive volunteers. For AMS-resistant volunteers, molecular pathways like activation of the complement system, and acute-phase response signaling are predicted to be activated in IPA-based pathway mining, LXR/RXR activation, coagulation system, and intrinsic prothrombin activation pathway. These results reinforce that suppression of energy-generating pathways and energy-consuming protein translation and elongation pathways play a crucial role during the early phase of high-altitude acclimatization.

The chronic exposure (days to weeks) strategy has been used to study the acclimatization response of lowlanders to high altitude [27, 59, 74, 104]. In order to identify serum biomarkers for high-altitude acclimatization at sea level, Yang et al. compared serum peptidome of high-altitude acclimatized (LLQ ≤ 3) and ill (LLQ ≥ 3) lowlanders [104] prior to high-altitude exposure. Using a comparative analysis of serum peptides at sea level, the authors have identified two upregulated serum peptide peaks (m/z values: 1061.9 and 1088.33 and one downregulated serum peptide peak (m/z value: 4057.63 corresponding to regions of proteins inter-α trypsin inhibitor heavy chain H4 (ITIH4, inter-α trypsin inhibitor heavy chain H1 fragment (ITIH1, and isoform 1 of fibrinogen α chain precursor (FGA, respectively, for high-altitude acclimatized lowlanders as compared to ill lowlanders. Subsequent ELISA-based studies also corroborated significant higher serum levels of ITIH1 (range 3.69–5.0 ng/mL and ITIH4 (range, 4.82–6.64 ng/mL in high-altitude acclimatized group as compared to ill group at sea level. ITIH1 and ITIH4 belong to inter-alpha-trypsin inhibitors (ITI, a family of plasma protease inhibitors contributing to extracellular matrix stability by covalent linkage to hyaluronan [15]. They accumulate in the vascular endothelium and might have a role in stabilizing endothelial cells and ECM damaged by high-altitude hypoxia. The third protein, fibrinogen, is a plasma glycoprotein that participates in the final phase of blood coagulation. Epidemiological studies have demonstrated that increased circulating levels of fibrinogen are a significant risk factor for cardiovascular disease, and fibrinogen biosynthesis is upregulated during hypobaric hypoxia [17, 95]. The studies of Padhy et al. and Gangwar et al. reported plasma proteome alterations of healthy lowlanders at high altitude (3500 m after 1, 4, and 7 days of exposure [27, 74]. These two studies have reported upregulation of acute-phase response proteins (SAA, AGP2, IGFBP1, ORM1, APCS, FGA, FGB, and FGG), complement pathway proteins, and inflammatory proteins (CRP, S100A8, and S100A9) in acclimatizing lowlanders. In addition, the authors have identified altered expression of several apolipoproteins (APOAI, APOAII, APOAIV, APOAV, APOB, APOCIII, APOCIV, APOD, APOE, APOH, APOL, and APOM) and associated lipoprotein pathway proteins (LCAT, CETP, and PLTP). Interestingly, the acclimatization process also results in higher plasma levels of KNG (kininogen), the precursor protein of the KKS (kallikrein–kinin system) and the KKS pathway proteins plasma KLKB1 (kallikrein) and BK (bradykinin). Activation of KKS pathway and resultant higher levels of BK modulates eNOS-mediated enhanced NO production promoting vasodilation and oxygen delivery, a process critical for hypoxia acclimatization [75]. In contrast, decreasing plasma ANGT (angiotensinogen) levels on day 7 compared to day 1 and day 4 of high-altitude exposure also supports activation of the vasodilation process during high-altitude acclimatization [27].

To understand the effect of prolonged exposure (3 months) to high altitude on lowlanders, Pooja et al. used a TMT (Tandem mass tags)-based proteomics approach to decipher plasma proteome level alterations after 3 months of high-altitude exposure [60]. The authors identified higher plasma levels of several apolipoproteins like APOAII, APOB, APOCI, APOCIII, APOE, and APOL, promoting dyslipidemia. In contrast, lower plasma levels of cytoskeletal proteins like PROF-1, ACTG1, TLN1, MYH9, and ACTN1 were identified after prolonged exposure. The molecular pathways associated with the altered proteome include plasma lipoprotein assembly, chylomicron assembly, chylomicron remodeling, VLDL (Very low-density lipoprotein) clearance, plasma lipoprotein clearance, and chylomicron clearance.

We prepared a comprehensive list of proteins identified for each high-altitude exposure time point (9 h, 1 day, 4 days, 7 days, and 90 days) and subjected it to IPA-based pathway analysis (Additional file 1). It is important to note we identified LXR/RXR activation as the common pathway for all the exposure time points. LXRs (Liver X receptors) represent a subset of the nuclear receptor superfamily that is regulated by oxidized forms of cholesterol (oxysterols) and intermediate products of the cholesterol biosynthetic pathway [49, 50]. LXRs form obligate heterodimers with RXRs (Retinoid X receptors), which are members of the nuclear receptor superfamily that can be regulated by 9-cis-retinoic acid (9cRA) and long-chain polyunsaturated fatty acids. LXR/RXR heterodimers regulate their target genes by recognizing specific LXR-response elements (LXRE) consisting of two direct hexanucleotide repeats separated by four nucleotides [98]. LXR/RXR heterodimers induce the expression of genes that mediate cholesterol efflux from cells to bile [81]. Activation of the LXR/RXR pathway is essential for regulating cholesterol homeostasis in macrophages, which can accumulate massive amounts of cholesterol during disease settings such as atherosclerosis [93]. A recent plasma proteomics investigation has reported dyslipidemia in lowlanders during a long-term stay at a high altitude, further supporting LXR/RXR activation during hypobaric hypoxia [59]. We have also identified acute-phase response and nitric oxide and reactive oxygen species production in macrophage pathways for all high-altitude exposure time points except the 30-min time point. Activation of these pathways at high altitude indicates inflammation and concomitant increase in acute-phase proteins, which might have adverse implications [25].

#### Urine and saliva

HA exposure also modulates the urinary protein profiles depending upon the altitude of ascending low landers (Fig. 2, Table 1). Studying urinary proteome of HIGH CARE-2008 study volunteers at sea level, HA (3500 m, Namche Bazaar) and very HA (5400 m, Mount Everest base camp), Mainini et al. identified six peptides (m/z 1165, 1683, 1953, 2194, 4298, 4757) differentially expressed in hypobaric hypoxia at high or very HA compared to the sea level [69]. These peptides identified two proteins, uromodulin (UMOD) and a1-antitrypsin (A1AT), corresponding to m/z 1683 and 2194, and levels were significantly reduced at both the studied altitudes. It is noteworthy that higher serum A1AT level is a risk factor for HAPE. Even minor increases in levels of serum A1AT are associated with the development of arterial hypertension and increased cardiovascular disease [14, 77]. These studies suggest that the upregulation of A1AT inhibits the activity of the kallikrein–kinin system that promotes HA acclimatization and favors the renin–angiotensin system leading to systemic vasoconstriction and hypertension [74]. Hence, downregulation of uromodulin and A1AT probably promotes nitric oxide availability, and vasodilation promotes HA acclimatization in low landers (Additional file 1).

The saliva proteome holds an excellent promise for HA research as it is a noninvasive body fluid and can be effectively used in devising effective strategies for the screening of HA fitness and early diagnosis of HA-induced pathologies. The salivary proteome of lowlanders was studied at 13,700 ft after 7-day post-ascent using 2D-gel electrophoresis [47]. By comparing with sea level saliva proteome, the authors identified a higher abundance of apoptosis-inducing factor-2 (AIFM2), cystatin S (CST4), cystatin SN (CST1), and carbonic anhydrase 6 (CA6) proteins for healthy lowlanders at HA. In contrast, a lower abundance of glycolysis-associated protein alpha-enolase was observed, indicating curtailed glycolysis corroborating the plasma proteomics observations for HA [48]. In addition, a lower abundance of immunoglobulin receptor and prolactin-inducible protein was observed, further substantiating the utility of saliva proteins for HA research. Subsequently, using an iTRAQ-based proteomics strategy, the authors compared the sea level saliva proteome with HA (4420 m) exposed saliva proteomes after 7, 30, and 120 days of stay. A total of 67 proteins were identified from saliva, out of which 56 (4 upregulated and 52 downregulated), 46 (10 upregulated and 36 downregulated), and 48 (7 upregulated and 41 downregulated) proteins were identified for 7, 30, and 120 days of high stay, respectively. Using IPA-based pathway analysis, the authors identified LXR/RXR activation, acute-phase response signaling, and production of reactive oxygen species and nitric oxide in macrophages as significant pathways (Additional file 1). In corroboration with previous salivary proteomics study, the authors reported lower enolase and prolactin-inducible protein (PIP) levels in all the study groups, suggesting curtailed glycolysis even after 120 days of stay at HA. Most notably, saliva proteomics studies also identified LXR/RXR pathway activation, corroborating plasma proteomics studies (Additional file 1). In contrast, the molecular production pathway of reactive oxygen species and nitric oxide in macrophages was downregulated across all exposure time points (days 7, 30, and 120). These studies also add to the growing consensus that lipid and energy are the key regulators of high-altitude acclimatization.

#### Muscle

Hypoxia exposure can have opposite effects on the regulation of muscle mass according to both degrees of hypoxia and exposure duration [22]. Macroscopic and microscopic muscle measurements after a sustained (8–12 weeks) sojourn at altitudes above 5,000 m demonstrated a reduction in lean lower limb mass of 10–15%, accompanied by a 20–25% decrease in mitochondrial volume density [36]. These morphological changes are associated with a proportional drop in muscle oxidative-enzyme activity and a relatively small reduction in glycolytic enzyme activities [37, 39, 61].

To investigate lowlanders muscle adaptation to HA, Vigano A et al. studied the muscle proteome after 7–9 days of exposure to HA (Margherita Hut, 4559 m) using 2D DIGE and MS. Separating muscle proteins on 4–7 and 6–11 pH range gels, the authors could identify altered abundance in 122 protein spots differentially changed in all subjects, among which 89 were decreased, and 33 were increased [94]. Out of these spots, 56 proteins were identified by MS, in which the abundance of 54 proteins was decreased while increased for 2. Further analysis revealed that proteins involved in iron transport (Serotransferrin), tricarboxylic acid (TCA) cycle, oxidative phosphorylation (2-oxoglutarate dehydrogenase (ODGH2), malate dehydrogenase (MDH1), and aconitate hydratase (ACO2), and oxidative stress responses (superoxide dismutase 1 (SOD1), glutathione S-transferase P (GSTP1), glutathione S-transferase Mu 2 (GSTM2), glutathione S-transferase Omega (GSTO1), peroxiredoxin 2 (PRDX2), peroxiredoxin 6 (PRDX6), protein deglycase 1 (DJ-1), cytoplasmic carbonic anhydrase 3 (CA-III), heat-shock protein beta 1 (HSPβ1), mitochondrial heat-shock protein 60 (HSP60) were significantly decreased during hypoxia exposure.

These results were further substantiated by independent research groups studying human muscle response to simulated hypoxia (Fraction of inspired oxygen, FiO_2_ -14.1%, 3,200 m for 15 days) and the Mount Everest Expedition (19 days at 5300 m altitude and 66 days up to 8848 m) [61, 21, 62]. Chronic exposure to hypobaric hypoxia results in an average 30% drop in muscular creatine kinase and glycolytic enzyme abundance. Similarly, the protein abundance of most enzymes of the tricarboxylic acid cycle and oxidative phosphorylation was reduced at HA, and the decrease was more evident at extreme altitudes. The elongation factor-2 alpha levels decrease at HA, indicating decreased protein translation, whereas increased heat-shock cognate 71 kDa protein levels were observed, indicating chaperone-mediated autophagy. HA exposure also damaged sarcomere structures, evidenced by increased protein levels of catalase and biliverdin reductase and decrement of voltage-dependent anion channels 1 and 2 and of myosin-binding protein C [19]. Chronic exposure of lowlanders to extreme altitude (6400 m and beyond) resulted in a decrease in muscle mitochondria density with loss of almost three fourth subsarcolemmal mitochondria in contrast to native Tibetans. Correspondingly, lower levels of the transcriptional coactivator PGC-1α also suggest downregulation of mitochondrial biogenesis with a concomitant decrease in expression of electron transport chain complexes I (NADH coenzyme Q oxidoreductase) and IV (cytochrome C oxidase) and mitochondrial uncoupling protein 3 (UCP3) levels [61]. Our IPA-based analysis identified the downregulation of glycolysis and gluconeogenesis pathways in muscle proteome during prolonged exposure (16 days and above) to extreme altitude. Our analysis also identified downregulation of HIF1α (hypoxia-inducible factor 1, Alpha) signaling pathway, sirtuin signaling pathway, and mitochondrial dysfunction across all exposure time points (Additional file 1). Our present observations corroborate that prolonged exposure of lowlanders to extreme altitudes decreases muscle energy-generating pathways, protein synthesis, mitochondrial biogenesis, and increased autophagy leading to muscle loss.

### Proteomic signatures for indigenous highlanders

**Fig. 1:**
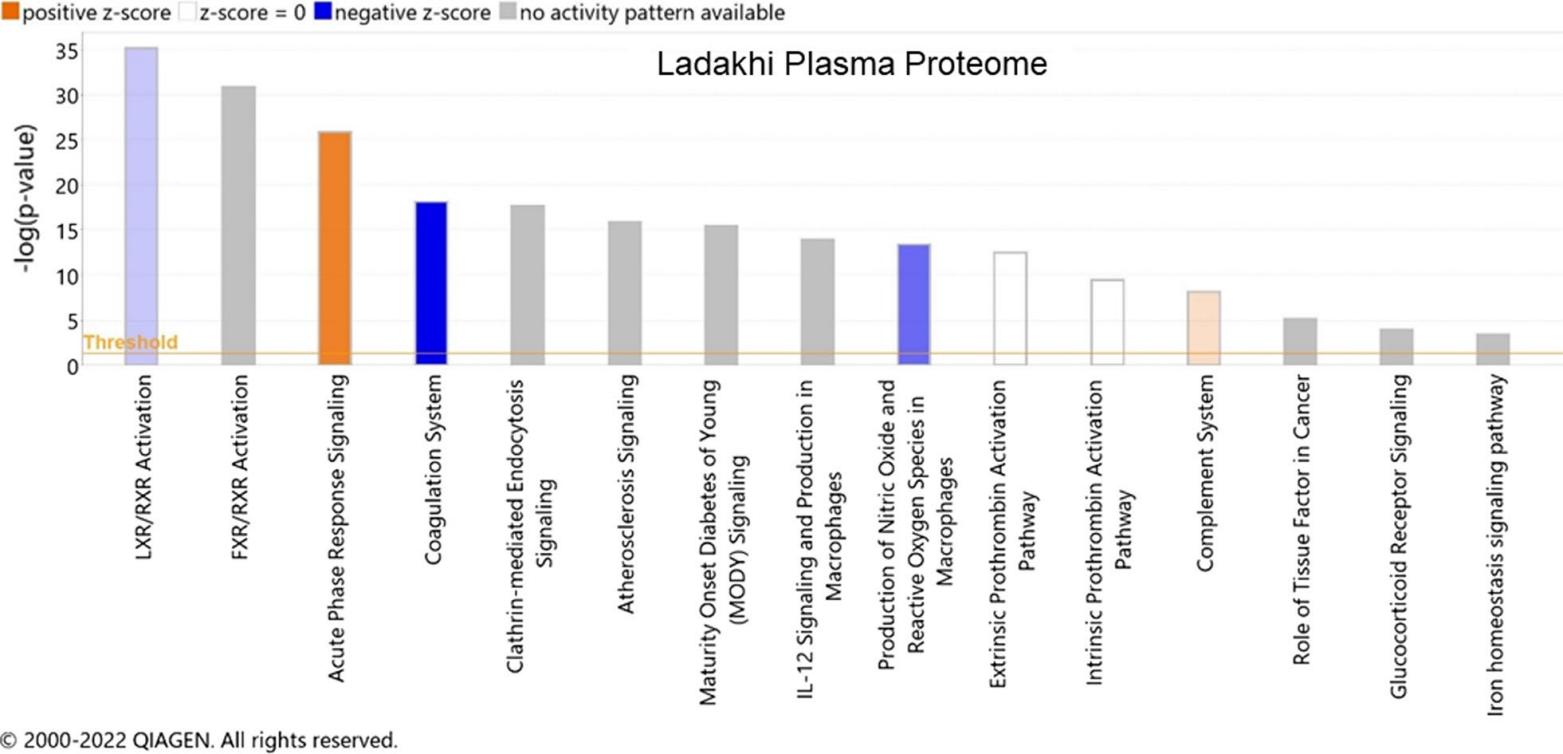
Top 15 Canonical pathways associated with differentially regulated plasma proteins identified for high-altitude native Ladakhi population. Pathway mining was performed using Ingenuity Pathway Analysis tool (https://www.qiagen.com/ontent-databases/ingenuity-pathway-analysis/)

A limited number of proteomics investigations have been performed to decipher plasma proteome alterations in Tibetan and Ladakhi highlanders [20, 75]. Using a TMT-based proteomics approach, Du et al. investigated alterations of Tibetan plasma proteome and identified changes in 137 proteins. The authors identified higher levels of CCL18, C9, PF4, MPO, and S100A9 while the levels of HRG and F11 decreased. These altered proteins participated in molecular functions like complement and coagulation cascades, antioxidative stress, and glycolysis [20] (Fig. 1). Investigating plasma proteome alterations of Ladakhi highlanders, Padhy et al. reported alterations in 36 plasma proteins belonging to blood pressure regulation (TETN, FIBB, AMBP, A2MG, FIBG, ANT3), acute-phase response (ITIH4, FETUA, ANGT, AACT), complement activation (C3, C4A, C1S), lipoproteins (APOE, APOM, APOL1, APOAIV), antioxidant proteins (A1BG, GPX3) and iron transport proteins (TRFE) [76]. The study identified lower plasma levels of angiotensinogen (ANGT) and angiotensin 2 (ANG II) with concomitant high levels of NO metabolites facilitates high-altitude adaptation of indigenous Ladakhi highlanders.

**Fig. 2:**
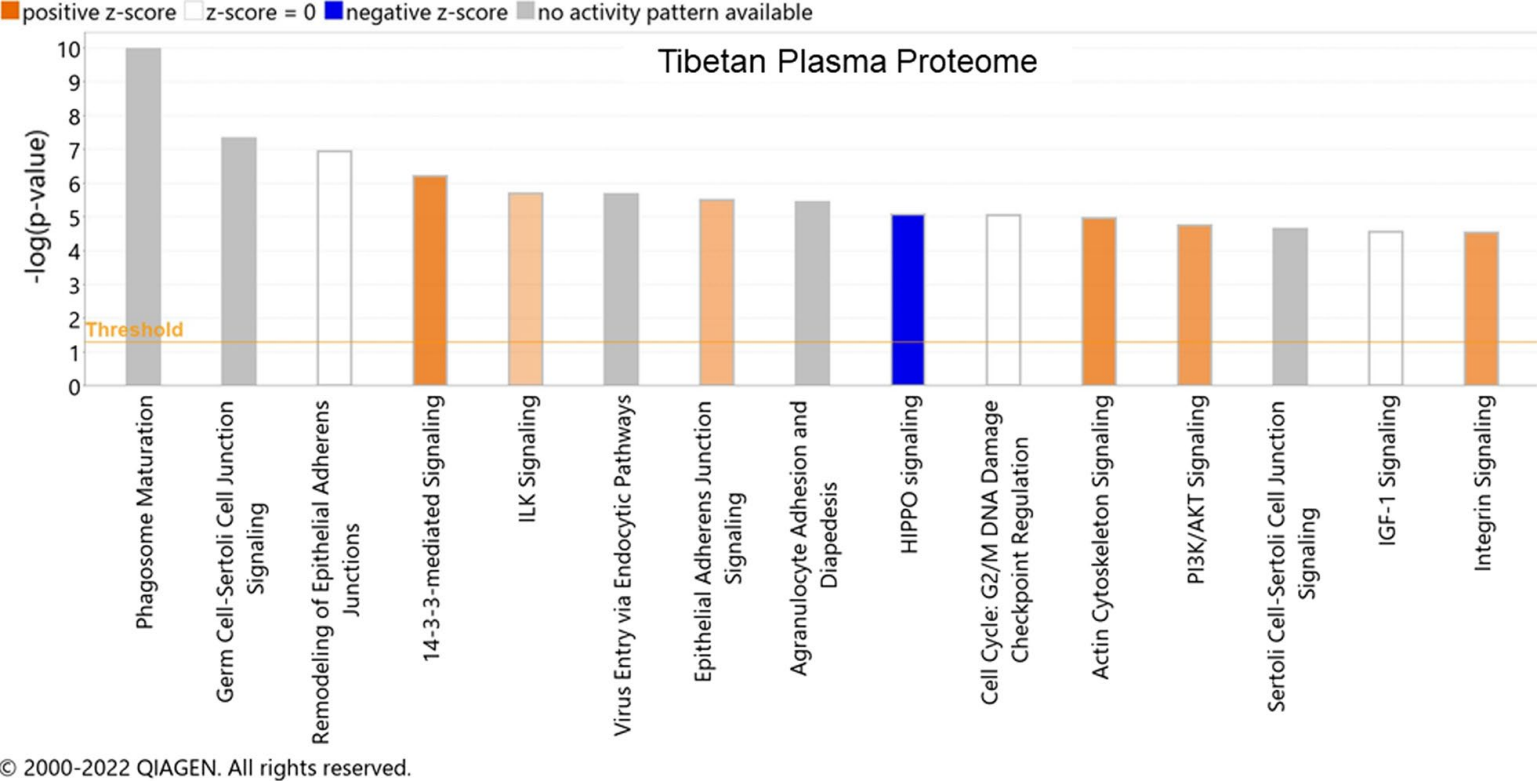
Top 15 Canonical pathways associated with differentially regulated plasma proteins identified for high-altitude native Tibetan population. Pathway mining was performed using Ingenuity Pathway Analysis tool (https://www.qiagen.com/ontent-databases/ingenuity-pathway-analysis/)

There is only one report on muscle proteomics on Tibetan highlanders. Gelfi et al. investigated the alterations in muscle proteome of native Tibetan highlanders permanently living at high altitude with second-generation Tibetans born at living at low altitude [29]. The authors also used muscle samples of lowland Nepal volunteers as a control. Using 2D-gel electrophoresis and mass spectrometry, the authors identified alterations in 7 proteins GSTP1-1, ECH (Enoyl CoA Hydratase), GAPDH, LDH, PGAM2, NADH ubiquinone oxidoreductase, and myoglobin. Lower levels of glycolytic proteins GAPDH and LDH were observed for Tibetan highlanders. In comparison, 400% higher levels of ECH, a member of fatty acid beta-oxidation pathway, NADH ubiquinone oxidoreductase, a member of mitochondria respiratory chain, and 380% higher levels of GSTP1-1was observed. These results indicate that native Tibetan highlanders are protected from ROS-induced tissue damage and have developed specific metabolic adaptations in the muscle tissue (Fig. 2).

### Genomic signatures for human adaptation to high altitude

Populations residing in three major high-altitude regions have been studied for population-specific genomic adaptation signatures: The Himalayas, including the Tibetan plateau, the Andean Altiplano, and the Ethiopian Highlands. Recent single-nucleotide polymorphism and whole-genome sequencing studies combined with statistical methods for detecting evidence of natural selection have compared genomes of high-altitude populations with similar low-altitude populations in an attempt to identify the genomic regions, genes, and genetic variants that exhibit signals of positive selection [12, 85]. Interestingly, the genes identified for these populations to date majorly belong to oxygen-sensing pathways under the regulation of hypoxia-inducible factor (HIF). In contrast, several other genes involved in the regulation of vascular control, metabolic hemostasis, redox homeostasis, and erythropoiesis, in general, have also been identified. In the present study, we have catalogued all the genes reported to date for experiencing positive selection.

#### Himalayan signatures

Studies comparing high-altitude Himalayan genomes (Tibetan and Sherpa) with matched low landers have identified a positive selection of *EPAS1* (endothelial PAS domain protein 1), which encodes the *HIF-2α* subunit of HIF complex, and *EGLN1,* which encodes *PHD2*, members of the HIF-oxygen-sensing pathway [9, 12, 85, 105]. Population-based genetic studies have identified adaptive evolution of *EPAS1* and *EGLN1* sequence variants that were significantly associated with the decreased hemoglobin phenotype, which is unique to Tibetans and Sherpas as compared to Andeans [9, 51, 52, 64, 78, 85, 102, 103]. Notably, an EPAS1 haplotype whose frequency is strongly correlated with altitude in the Himalayan populations has been suggested to have been acquired from an extinct hominin species, Denisovans [32, 43]. Studying polygenic adaptation of high-altitude Himalayan populations, Gnecchi-Ruscone et al. have reported a positive selection of many genes, including COL11A1, COL11A2, ESR1 *LAMC1*, *LAMC2*, *ITGA6*, *ITGA1* and *ITGA2* in Tibetans and Sherpas [30]. Multiple studies examining of positive selection of Himalayan populations, including Tibetan, Sherpa, and Nepalese genomes for hypoxia adaptation, have reported candidate gene loci like *PPARA*, *HBB*, *MTHFR*, *SLC52A3*, *ANKH*, *ZNF532*, *COL4A4*, *MKL1*, and *GRB2* genes to name a few (Additional file 1: Fig. S4, Table S2), [3, 4, 103] (Additional file 1).

#### Andean signatures

Population genetics investigations for native Andean highlanders have identified positive selection for several genes, including *EDNRA*, *PRKAA1,* and *NOS2A,* along with *EGLN* [12, 13]. Using whole-genome sequencing of Andeans, Crawford et al. have identified a positive selection of multiple genes, including *BRINP3*, *NOS2,* and *TBX5,* associated with cardiovascular development and function, unlike Tibetans [18]. In corroboration, a study of whole ancient genomes from the Andes of Peru, dating back to 7,000 calendar years before the present (BP), has identified a positive selection of *DST,* a gene involved in cardiovascular function and *MGAM* related to starch digestion and plausibly pathogen resistance after European contact [63] (Additional file 1).

These studies suggest that positive selection in the Andes has focused on the nitric oxide pathway and cardiovascular system, unlike the HIF pathway of Himalayan highlanders. Using the candidate gene approach, Jacovas et al. have reported alleles of *TP53* genes, namely *USP7*, *LIF,* and *MDM2,* selected for the successful establishment of Native American populations in the Andean highlands [46]. In a follow-up study, the authors have identified a positive selection of *TP53* pathway genes *SP100*, *DUOX2,* and *CLC,* imparting physiological benefits like increased angiogenesis, skeletal muscle adaptations, and immune functions for Andean highlanders [45]. Multiple studies comparing Andean highlanders with and without chronic mountain sickness further identified additional genes that seem to protect against chronic disease at high altitude, including *ANP32D*, *SENP1*, *PRDM1*, *AEBP2*, *CAST,* and *MCTP2* (Additional file 1: Fig. S4, Table S2), [28, 89, 106] (Additional file 1).

#### Ethiopian signatures

Ethiopian highlanders are the least studied population for their adaptations to high altitude because they maintain similar hemoglobin concentrations and arterial oxygen saturation within the ranges compared to sea level populations, despite persistent hypobaric hypoxia [10]. In addition, Ethiopian highlanders are not isolated populations like Tibetans and show evidence of gene flow outside Africa. A genomic analysis of high-altitude Amhara populations in Ethiopia has identified candidate genes CBARA1, VAV3, ARNT2, and THRB, distinct from those identified in Andeans and Tibetans. Interestingly, two genes (*THRB* and *ARNT2*) are part of the HIF-mediated oxygen-sensing pathway indicating independent convergent evolution of hypoxia adaptation responses [83]. A comparative analysis of two ethnic populations, Amhara and Oromo, identified a selection of hypoxia-associated genes, including *RORA* (RAR-related orphan receptor A) in the Amhara. They have lived at high altitudes for thousands of years compared to the Oromo, with 500 years of high-altitude inhabitation history.

Other strong associations were observed in other candidate hypoxia genes, such as *COL6A1*, *SLC30A9,* and *HGF* [2]. Subsequent comparative evaluation of single-nucleotide polymorphism genotyping data from five Ethiopian populations (three of which are high altitude) has identified the strongest signal of selection in BHLHE41, which regulates the hypoxia response pathway in both Amhara and Oromo populations [42]. Whole-genome sequencing approach has identified three more genes, *CIC*, *LIPE,* and *PAFAH1B3,* with a subsequent demonstration of increased hypoxia tolerance in the orthologous genes in Drosophila [92]. A more recent study examining the polygenic adaptation of 119 genomes constituting five populations has identified the selection of folate metabolism (*FOLR1*, *FOLR2,* and *DHFRL1*) and the related ultraviolet response and skin pigmentation genes (*UVRAG* and *BNC2*) to cope with high levels of ultraviolet irradiation. In addition, the selection was also observed for genes such as *IFNA* and *MRC1,* which contribute to defending against pathogens [96]. A total of 163 positively selected gene signals are reported in previously published literature. These include gene signals identified in Himalayan, Andean, and Ethiopian high-altitude populations (Additional file 1).

### Convergence of genomic and proteomic signatures

**Fig. 3:**
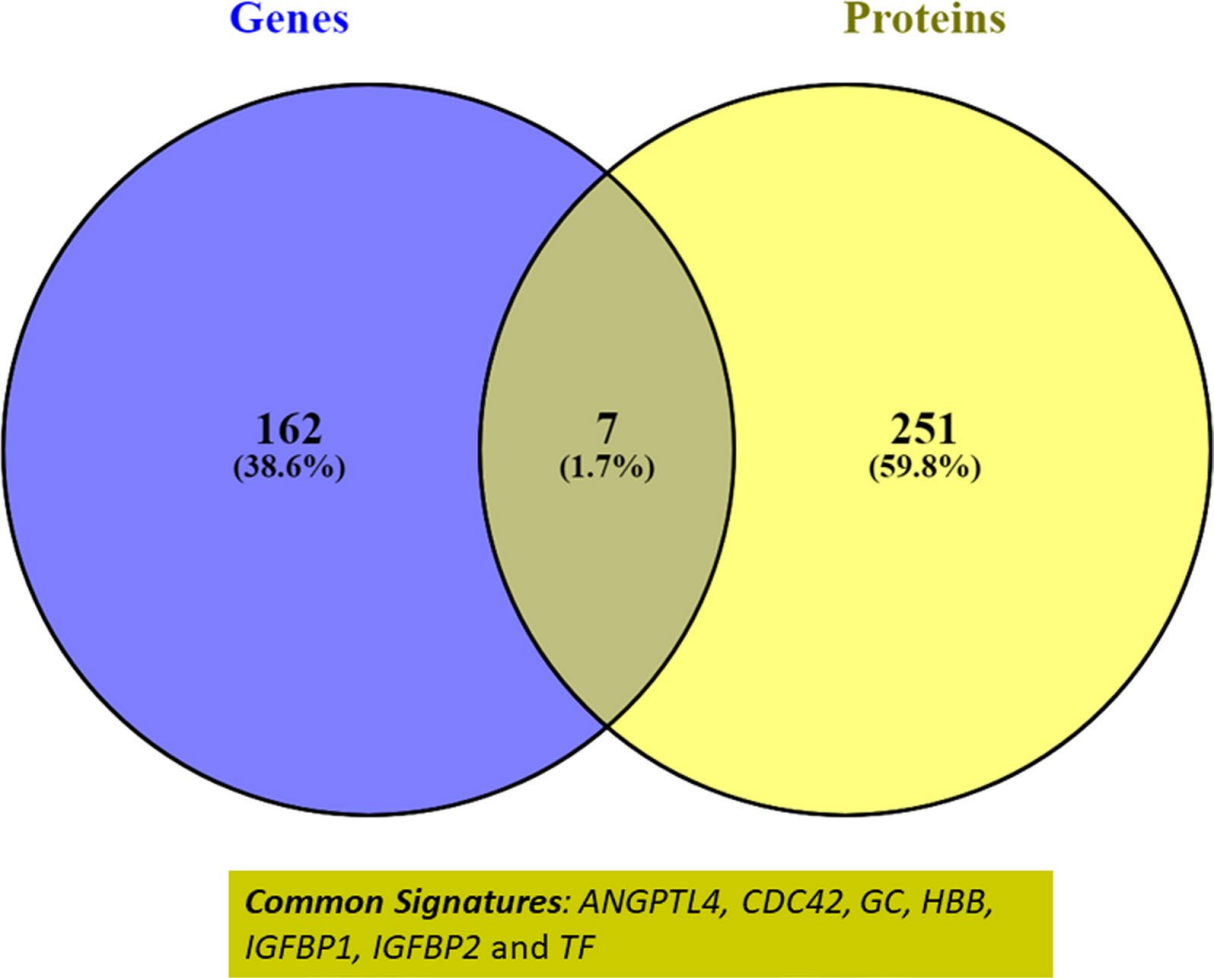
A comparison of the genetic selection signatures which impart selective advantage in hypoxic environment at high altitude and protein markers differentially expressed in Tibetan/Sherpa, Ladakhis, Andean populations, and Ethiopian high-altitude populations. A total of seven genes/proteins namely *HBB, TF, ANGPTL4, CDC42, GC, IGFBP1*, and *IGFBP2* have been identified as convergent signatures for high-altitude adaptation

Genomics studies for geographically diverse HA populations over the past decade have identified 169 genes under positive selection (Additional file 1). The top GO molecular functions identified for these genes include oxygen binding, receptor binding, iron-binding, and VEGF receptor binding. Similarly, proteomics-based studies have identified 258 proteins from biofluids and muscle tissue, corroborating genomics studies. Analysis of similarity between genomics and proteomics signatures resulted in the identification of seven common members, namely *HBB*/Hemoglobin subunit beta*, TF*/Transferrin*, ANGPTL4*/Angiopoietin-related protein 4*, CDC42*/Cell division control protein 42 homolog*, GC*/Vitamin D-binding protein*, IGFBP1*/Insulin-like growth factor-binding protein1 and *IGFBP2*/Insulin-like growth factor-binding protein 2) (Fig. 3). Hemoglobin subunit beta is involved in oxygen transport from the lung to the various peripheral tissues. Transferrin is a 76.5 kDa glycoprotein involved in the transport of Fe (3 +) ions in association with bicarbonate ions. This protein is responsible for the transport of iron from sites of absorption and heme degradation to those of storage and utilization. Proteomics studies for Ladakhi natives also identified lower TF and higher levels of GC though vitamin D insufficiency is prevalent in diverse, high-altitude populations [1, 56]. Lowlanders ascending to high altitude also exhibit reduced levels of 25(OH)D compared to pre-ascent levels. These changes were associated with the modulation of immune response [57]. Transferrin levels generally decrease upon ascent to high altitude irrespective of ethnicity, possibly due to augmented erythropoiesis [68]. ANGPTL4 is a secreted glycoprotein induced by peroxisome proliferation activators and plays a physiological role in lipid and glucose metabolism [86]. ANGPTL4 inhibits the activity of lipoprotein lipase (LPL) and thereby promotes an increase in circulating triglyceride levels [24]. Insulin-like growth factor (IGF)-binding protein (IGFBP)-1 is a liver secreted plasma protein that binds to IGF in extracellular environments. Elevated levels of IFFBP1 protein have been reported in maternal serum at high altitude and in intrauterine growth restriction (IUGR) fetuses [54, 58]. There is a striking inverse correlation between IGFBP1 and IGFBP2 levels [23, 90], with intrauterine fetal growth restriction and size indicating its importance for high-altitude adaptation. Our IPA-based network analysis also identified molecular pathways like IGF-1 signaling, LXR/RXR activation, ferroptosis signaling, iron homeostasis signaling, and regulation of cell cycle for these common members.

There are several reasons for identifying only seven common members from the genomics and proteomics dataset. First, genomics studies were targeted at identifying genomic regions, genes, allele variants, and SNPs under positive natural selection. Many of these alleles or SNPs may not have a functional protein or tissue-specific expression and have not been identified. In contrast, most proteomics studies identified proteins and specific biological pathways for understanding the acclimatization response of lowlanders from body fluids and muscle tissue. Second, the selection of high-altitude populations was mainly concentrated around genomic regions of oxygen sensing, transport, and delivery, along with protection from other altitude-associated environmental factors like high UV radiation and cold. In contrast, lowlanders were not subjected to such selective pressure. Third, high-altitude genomics studies regularly analyze the whole human genome, whereas proteomics investigations analyze only a few hundred proteins because of technical limitations. Fourth, high-altitude genomics studies have identified several transcription factors (*HIF1A* and *PPARA*) and associated genes that orchestrate oxygen-sensing pathways, whereas proteomics studies are limited in identifying such regulators. Fifth, the available proteomics techniques (protein separation and mass spectrometer-based identification) suffer from the limitations of identifying only a subset of proteins of the whole proteome. These observations warrant more detailed omics-based investigations to identify the specific gene or protein variants for the integrated adaptive response of humans to high altitude. Such information can help design better acclimatization protocols, screen individual susceptibility to high altitude, sports nutrition and performance, and better management of ICU-related illnesses.

## Future directions

High altitude is a natural laboratory affecting humans' birth, growth, and performance, permanently living at high altitudes and sojourners. The high-altitude regions have a long history of human inhabitation through geographically isolated populations that have developed unique adaptive features to a shared environment. Many genomic studies have highlighted the genes and variants responsible for specific adaptive patterns of Tibetans, Andeans, and some Ethiopians. More recently, *EPAS1* gene variants and their association with hemoglobin concentration, adaptive introgression of Denisovan allele, and high-altitude adaptation have also been extensively studied in Tibetans [40, 41, 43, 65, 78–80, 103].

Unfortunately, the functional role of other identified genomic signatures like activation of the immune response, digestion, and skin pigmentation is not fully established due to the complexity of traits at environment genotype interaction and other signaling mechanisms at the cellular and molecular level. It is important to note that other additional adaptive features would be required to cope with other environmental factors like cold, increased UV radiation, and limited availability of food resources. These responses may be further explored using gene-editing tools and gene expression assays to augment the understanding in high-altitude hypoxia research. Functional validation of the identified genes and proteomics-assisted biological pathway information for each trait and inter-individual differences of essential proteins can add more value to high-altitude research. These targeted studies should include other high-altitude populations like Sherpas, central Asian highlanders like Kyrgyz, and other indigenous populations of the Andean plateau.

Unlike genomics investigations, only a handful of global proteomics studies have been conducted to decipher acclimatization and adaptive high-altitude signatures. These limited proteomics studies have been restricted to circulatory biofluids like plasma, serum, urine, and muscle tissue, enabling the identification of only a few hundred proteins. Recent developments in protein separation techniques combined with new age mass spectrometers and detection techniques enable researchers to identify thousands of proteins from biological specimens within a few days [70, 87]. High-altitude biologists should utilize these proteomics techniques to identify many proteins to complement genomics studies. Such information will provide new insight into associations between genetic variants and protein abundance, providing insights undetectable by mRNA associations [6]. In addition, increased throughput will afford the analysis of a more significant number of samples, increasing statistical power [87]. As mentioned earlier, plasma proteomics studies have been conducted only for Tibetan and Ladakhi populations. No such proteomics signature is available for Andean and Ethiopian highlanders in literature, limiting the current understanding of functional adaption to high altitude.

It is noteworthy that high-altitude-responsive protein expression is dependent on environmental conditions (altitude of stay, duration of stay, cold and UV radiation exposure) and genotype interactions (intra-individual variations). In addition, all the laboratories use their protein isolation, separation, and labeling techniques, mass spectrometers with varied run conditions, and statistical parameters for protein identification. Differences in these parameters constrain the reproducibility of the experiments and the number of identified proteins [44, 84]. Hence, a joint global initiative for high-altitude-responsive protein identification project is the need of the hour. Such initiative covering diverse indigenous, high-altitude populations and lowlanders will complement the ongoing genomic initiatives for high-altitude adaptation and maladaptation.

## Supplementary Information

**Additional file 1** contains IPA based integrated analysis of temporal high altitude proteomics data and overlapping canonical pathways associated with common proteins and gene selection signaures of high altitude adaptation. 1. Figure S1 to S4 reperesent the top canonical pathways associated with high altitude proteomics data in temporal human plasma, saliva and serum samples using IPA. 2. Figure S5 represents the top 15 canonical pathways associated with the overlapping protein markers and gene selection signatures for high altitude adaptation. 3. Table S2 and S3 represents the identified gene selection signatures reported to be imparting the positive selection in HA environment and protein markers differentially regulated at high altitude.
