# Supplementary material for: Human adaptation to high altitude: a review of convergence between genomic and proteomic signatures
Source: Hum Genomics. 2022 Jul 15;16:21. doi: 10.1186/s40246-022-00395-y (PMC9287971; doi:10.1186/s40246-022-00395-y)
Supplement: Supplementary file 1 — Additional file 1 contains IPA based integrated analysis of temporal high altitude proteomics data and overlapping canonical pathways associated with common proteins and gene selection signaures of high altitude adaptation. 1. Figure S1 to S4 reperesent the top canonical pathways associated with high altitude proteomics data in temporal human plasma, saliva and serum samples using IPA. 2. Figure S5 represents the top 15 canonical pathways associated with the overlapping protein markers and gene selection signatures for high altitude adaptation. 3. Table S2 and S3 represents the identified gene selection signatures reported to be imparting the positive selection in HA environment and protein markers differentially regulated at high altitude. [file 40246_2022_395_MOESM1_ESM.docx]

**Additional File 1**

**Human response to high altitude: Analysis of convergence between genomic and proteomic signatures**

Vandana Sharma, Rajeev Varshney, Niroj Kumar Sethy*

Peptide and Proteomics Division, Defence Institute of Physiology and Allied Sciences (DIPAS), Defence Research and Development Organisation (DRDO), Lucknow Road, Timarpur, Delhi – 110054, India

* Address for correspondence:

Niroj K. Sethy, Ph. D.

Peptide and Proteomics Division,

Defence Institute of Physiology and Allied Sciences (DIPAS),

Defence Research and Development Organisation (DRDO),

Lucknow Road, Timarpur, Delhi – 110054, India

Phone: +91-11-23883003

Fax: +91-11-23917420

e-mail: niroj.dipas@gov.in

**Figure S1:** Identification of top 15 canonical pathways associated with lowlander plasma proteome after high altitude exposure of 9 hours, 1day and 4 days respectively.


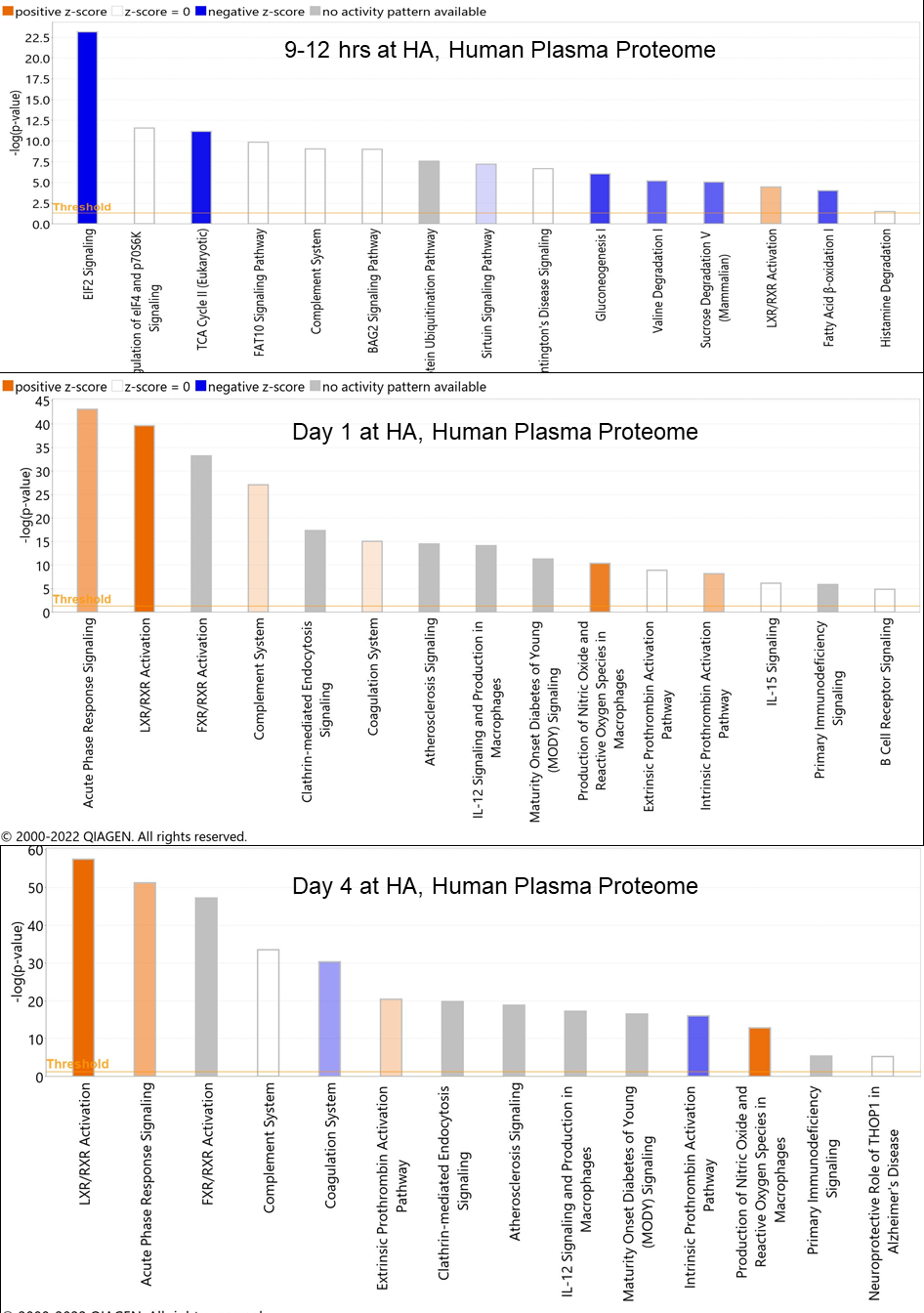


**Figure S2:** Identification of top 15 canonical pathways associated with lowlander plasma proteome after high altitude exposure 7 days and 3 months respectively.


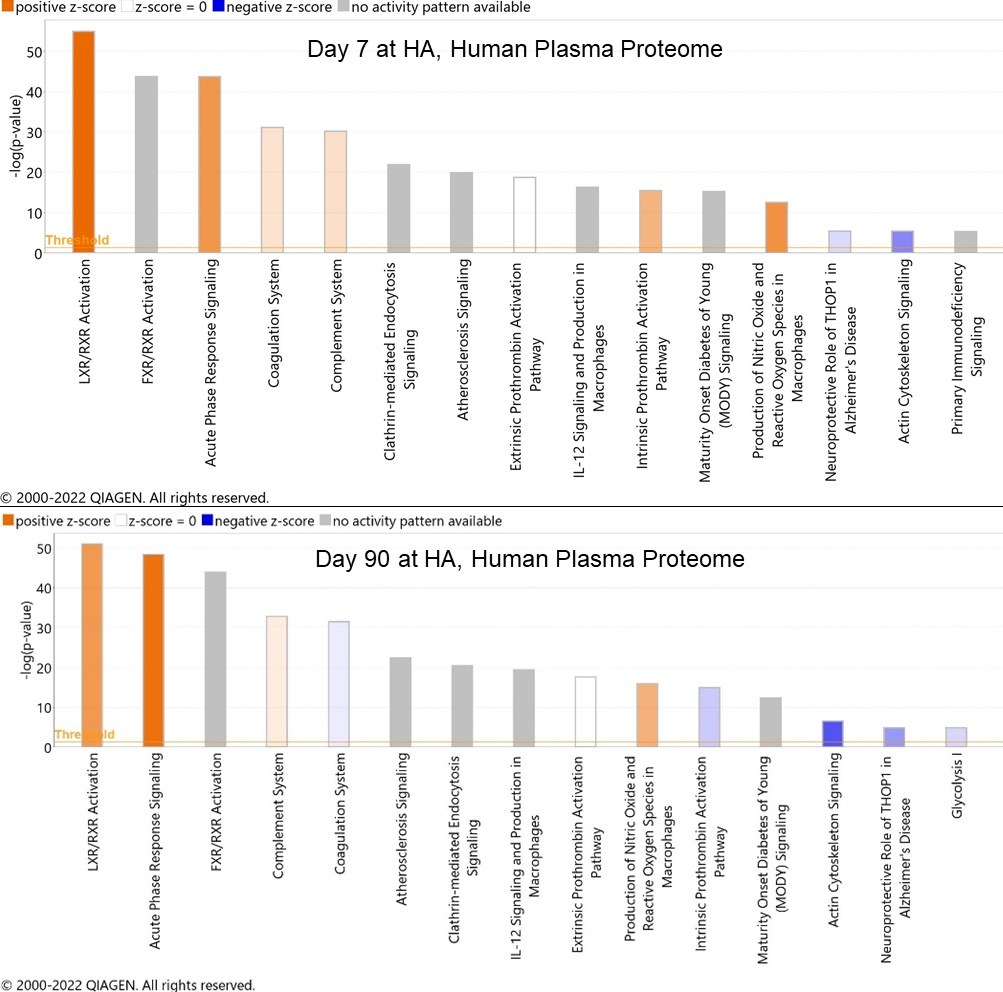


**Figure S3:** Identification of top 15 canonical pathways associated with lowlander saliva proteome after high altitude exposure of 7 days, 30 days and 120 days respectively.


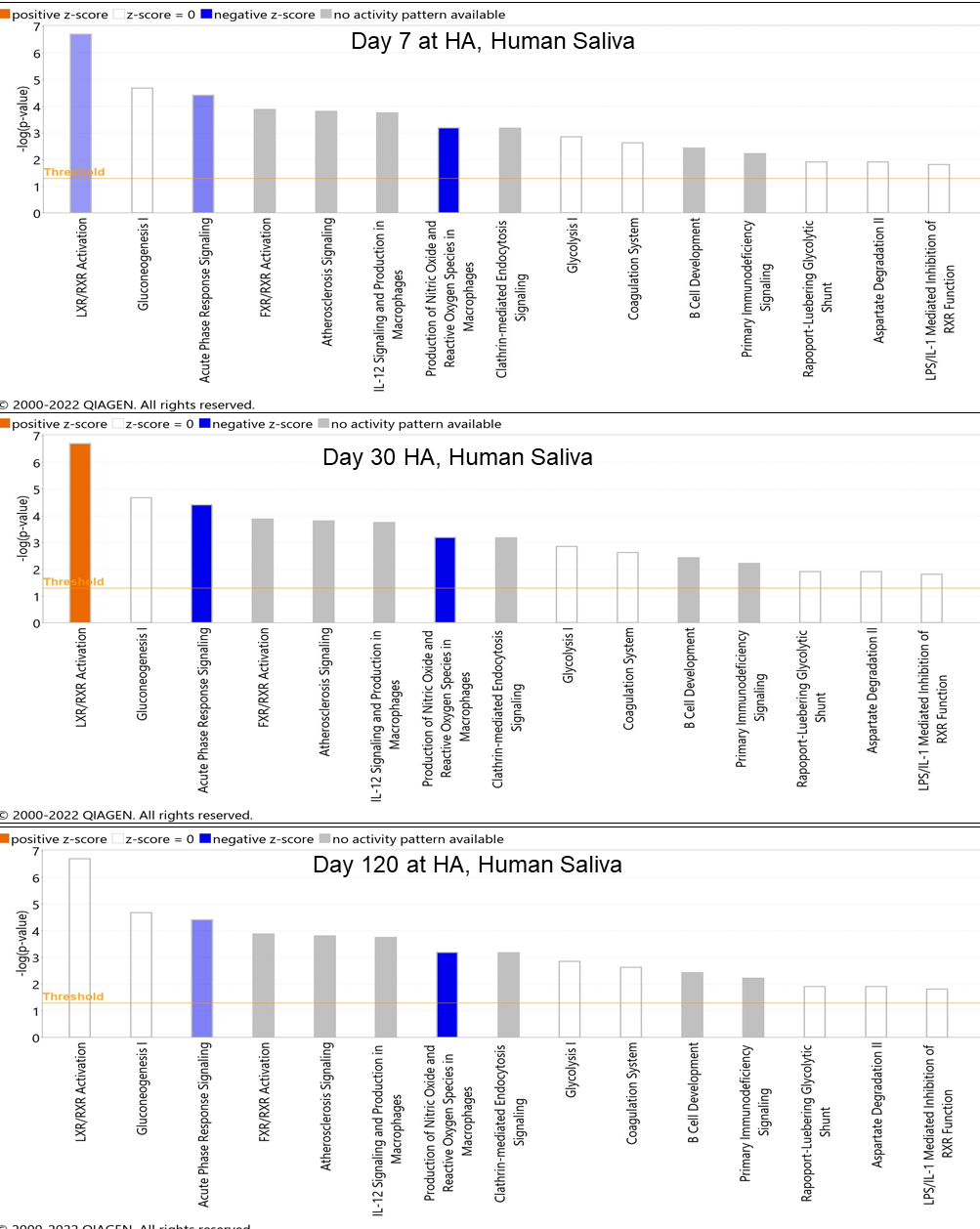


**Figure S4:** Identification of top 15 canonical pathways associated with lowlander muscle proteome after high altitude exposure of 7-9 days, 16-20 days and 2 months respectively.


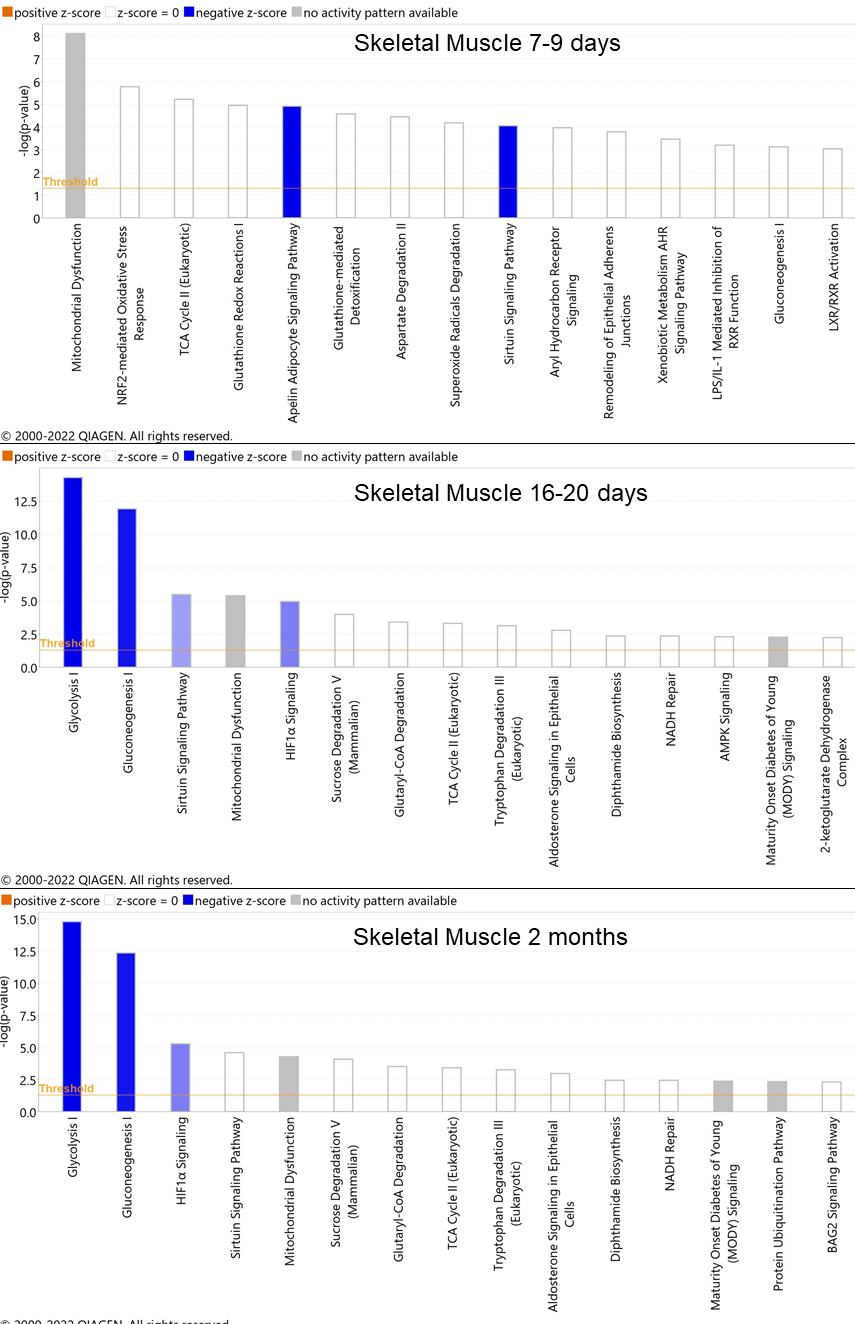


**Figure S5:** Top 15 Canonical pathways associated with convergent gene/protein signature for human response to high altitude adaptation (Tibetan/Sherpa and Ladakhi high altitude natives).


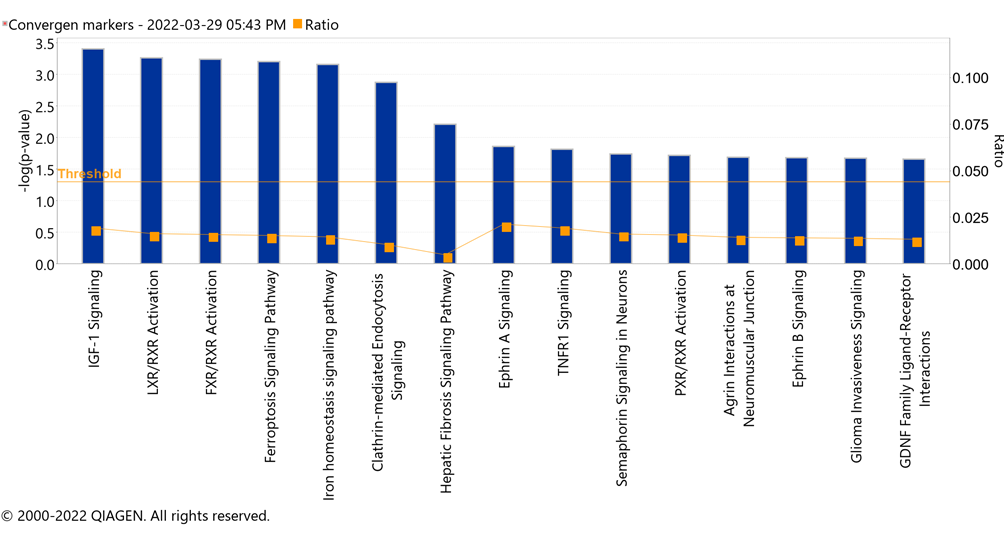


**Table S2: List of genes associated with positive natural selection at high altitude.** This table represents genes positively selected in high altitude Andean, Ethiopian and Himalayans, high-altitude populations.

| **Sr. no.** | **Gene** | **Protein encoded** | **Population** | **Reference** |
| --- | --- | --- | --- | --- |
|  | *AC068633.1* | - | Himalayan | ^19^ |
|  | *ACE* | Angiotensin I Converting Enzyme | Himalayan | ^20^ |
|  | *ADRA1B* | Alpha-1B Adrenergic Receptor | Andean/ Himalayan | ^21,22,23^ |
|  | *ADRBK1/GRK2* | G Protein-Coupled Receptor Kinase 2 | Ethiopians | ^24^ |
|  | *ALDH3A1* | Aldehyde Dehydrogenase 3 A1 | Himalayan | ^25^ |
|  | *ANGPT1* | Angiopoietin 1 | Himalayan | ^26^ |
|  | *ANGPTL4* | Angiopoietin Like 4 | Himalayan | ^22^ |
|  | *ANKH* | ANKH Inorganic Pyrophosphate Transport Regulator | Himalayan | ^19^ |
|  | *ARNT2* | Aryl Hydrocarbon Receptor Nuclear Translocator | Himalayan /Ethiopians/  Andeans | ^21,27, 28,23,29^ |
|  | *ASF1B* | Anti-Silencing Function 1B Histone Chaperone | Ethiopians | ^24^ |
|  | *BHLHE41* | Basic Helix-Loop-Helix Family Member E41 | Ethiopians | ^30^ |
|  | *C6orf195* | Long Intergenic Non-Protein Coding RNA 1600 | Himalayan | ^31^ |
|  | *CAMK2D* | Calcium/Calmodulin Dependent Protein Kinase II Delta | Himalayan | ^22^ |
|  | *CASP1* | Caspase 1 | Ethiopians | ^30^ |
|  | *CASR* | Calcium Sensing Receptor | Himalayan | ^21^ |
|  | *CBARA1/MICU* | Calcium Binding Atopy-Related Autoantigen 1 | Ethiopians | ^29^ |
|  | *CBS* | Cystathionine Beta-Synthase | Andean | ^32^ |
|  | *CCDC141* | Coiled-Coil Domain Containing 141 | Himalayan | ^31^ |
|  | *CDC42* | Cell Division Cycle 42 | Himalayan | ^33^ |
|  | *CDCA7L* | Cell Division Cycle Associated 7 Like | Himalayan | ^25^ |
|  | *CDH1* | Cadherin 1 | Andean | ^34^ |
|  | *CDH13* | Cadherin 13 | Himalayan | ^26^ |
|  | *CIC* | Capicua Transcriptional Repressor | Ethiopians | ^35^ |
|  | *CLC* | Charcot-Leyden Crystal Galectin | Andean | ^36^ |
|  | *CO4A4* | Collagen Type IV Alpha 4 Chain | Himalayan | ^19^ |
|  | *COL11A1* | Collagen Type XI Alpha 1 Chain | Himalayan | ^33^ |
|  | *COL11A2* | Collagen Type XI Alpha 2 Chain | Himalayan | ^33^ |
|  | *COL6A1* | Collagen Type VI Alpha 1 Chain | Himalayan | ^33^ |
|  | *CORO1B* | Coronin 1B | Ethiopians | ^24^ |
|  | *CTAGE1* | Cutaneous T Cell Lymphoma-Associated Antigen 1 | Andean | ^37^ |
|  | *CTBP2* | C-Terminal Binding Protein 2 | Himalayan | ^31^ |
|  | *CUL3* | Cullin 3 | Ethiopians | ^24^ |
|  | *CXCL17* | C-X-C Motif Chemokine Ligand 17 | Ethiopians | ^35^ |
|  | *CXCR4* | C-X-C Motif Chemokine Receptor 4 | Andean | ^23^  ^21^ |
|  | *CYP17A1* | Cytochrome P450 Family 17 Subfamily A Member 1 | Himalayan | ^22^ |
|  | *CYP2E1* | Cytochrome P450 Family 2 Subfamily E Member 1 | Himalayan | ^24^ |
|  | *DISC1* | DISC1 Scaffold Protein | Himalayan | ^38^ |
|  | *DLG* | Discs Large MAGUK Scaffold Protein 3 | Ethiopians | ^35^ |
|  | *DLG1* | Discs Large MAGUK Scaffold Protein 1 | Himalayan | ^25^ |
|  | *DST* | Dystonin | Andean | ^39^ |
|  | *DUOX2* | Dual Oxidase 2 | Andean | ^36^ |
|  | *EDAR* | Ectodysplasin A Receptor | Himalayan | ^25^ |
|  | *EDN1* | Endothelin 1 | Andean/ Himalayan | ^21,23^ |
|  | *EDNRA* | Endothelin Receptor Type A | Andean/ Himalayan | ^21,22,23^ |
|  | *EDNRB* | Endothelin Receptor Type B | Andean/Ethiopian | ^21,35^ |
|  | *EDRF1* | Erythroid Differentiation Regulatory Factor 1 | Himalayan | ^31^ |
|  | *EGLN1* | Prolyl hydroxylase 2 | Andean/ Himalayan | ^40, 22, 21, 41,23,42,43^ |
|  | *EGLN2* | Egl-9 Family Hypoxia Inducible Factor 2 | Andean | ^21^ |
|  | *ELF2* | E74 Like ETS Transcription Factor 2 | Andean | ^21^ |
|  | *ELTD1/ADGRL4* | Adhesion G Protein-Coupled Receptor L4 | Andean | ^32^ |
|  | *EP300* | E1A Binding Protein P300 | Himalayan | ^40^ |
|  | *EPAS1* | Endothelial PAS Domain Protein 1 | Andean/ Himalayan | ^22,21,38,44,34^ |
|  | *EPO* | Erythropoietin | Himalayan /Andean | ^21,44^ |
|  | *ESR1* | Estrogen Receptor 1 | Himalayan | ^33^ |
|  | *ET1* | Endothelin 1 | Andean/Ethiopians | ^45^ |
|  | *FAM213A* | Family with Sequence Similarity 213 Member A | Andean | ^46^ |
|  | *FANCA* | FA Complementation Group A | Himalayan | ^38^ |
|  | *FLT1* | Fms Related Receptor Tyrosine Kinase 1 | Himalayan | ^21^ |
|  | *FOXO1* | Forkhead Box O1 | Himalayan | ^26^ |
|  | *GC* | Vitamin D-binding protein | Himalayan | ^47^ |
|  | *GCH1* | GTP Cyclohydrolase 1 | Himalayan | ^48^ |
|  | *GRB2* | Growth Factor Receptor Bound Protein 2 | Himalayan | ^19^ |
|  | *HBB* | Hemoglobin beta chain | Himalayan | ^40,22,38^ |
|  | *HBG2* | Hemoglobin Subunit Gamma 2 | Himalayan | ^40,38^ |
|  | *HEART5B* |  | Himalayan | ^25^ |
|  | *HGF* | Hepatocyte Growth Factor | Ethiopians | ^24^ |
|  | *HIF1A* | Hypoxia inducible factor 1 alpha | Himalayan /Andean | ^40,22,21,38^ |
|  | *HLA-DOB1* | - | Himalayan | ^19^ |
|  | *HMBS/HYOUI* | Hydroxymethylbilane Synthase | Himalayan | ^49^ |
|  | *HMOX2* | Heme Oxygenase 2 | Himalayan | ^22^ |
|  | *IGFBP1* | Insulin Like Growth Factor Binding Protein 1 | Andean/ Himalayan | ^21,23^ |
|  | *IGFBP2* | Insulin Like Growth Factor Binding Protein 2 | Andean/ Himalayan | ^21,23^ |
|  | *IL1A* | Interleukin 1 Alpha | Andean/ Himalayan | ^21,23^ |
|  | *IL1B* | Interleukin 1 Beta | Andean/ Himalayan | ^21,23^ |
|  | *IL6* | Interleukin 6 | Andean/ Himalayan | ^21,23^ |
|  | *ITGA1* | Integrin Subunit Alpha 1 | Himalayan | ^33^ |
|  | *ITGA2* | Integrin Subunit Alpha 2 | Himalayan | ^33^ |
|  | *ITGA6* | Integrin Subunit Alpha 6 | Himalayan | ^33^ |
|  | *KCNMA1* | Potassium Calcium-Activated Channel Subfamily M Alpha 1 | Andean/ Himalayan | ^21,23,34^ |
|  | *KCTD12* | Potassium Channel Tetramerization Domain Containing 12 | Himalayan | ^47^ |
|  | *KIAA0125* | Family with Sequence Similarity 30 Member A | Ethiopians | ^35^ |
|  | *LAMC1* | Laminin Subunit Gamma 1 | Himalayan | ^33^ |
|  | *LAMC2* | Laminin Subunit Gamma 2 | Himalayan | ^33^ |
|  | *LEPR* | Leptin Receptor | Himalayan | ^26^ |
|  | *LIF* | LIF Interleukin 6 Family Cytokine | Andean | ^50^ |
|  | *LIPE* | Lipase E, hormone sensitive type | Ethiopian | ^34^ |
|  | *MAPKAPK2* | MAPK Activated Protein Kinase 2 | Ethiopians | ^24^ |
|  | *MARCH8* | Membrane Associated Ring-CH-Type Finger 8 | Himalayan | ^25^ |
|  | *MDM2* | MDM2 Proto-Oncogene | Andean/ Himalayan | ^21,23,50^ |
|  | *MGAM* | Maltase-Glucoamylase | Andean | ^39^ |
|  | *MKL1* | MKL/Myocardin-Like Protein 1 | Andean | ^19^ |
|  | *MLK1* | Mitogen-Activated Protein Kinase Kinase Kinase 9 | Himalayan | ^51^ |
|  | *MMP2* | Matrix metalloprotease 2 | Andean/ Himalayan | ^21,23,26^ |
|  | *MTHFR* | Methylenetetrahydrofolate Reductase | Himalayan | ^28^ |
|  | *MTOR* | Mechanistic Target of Rapamycin Kinase | Andean | ^21,23^ |
|  | *MYB* | MYB Proto-Oncogene, Transcription Factor | Himalayan | ^33^ |
|  | *MYC* | MYC Proto-Oncogene, BHLH Transcription Factor | Himalayan | ^33^ |
|  | *NOS1* | Nitric Oxide Synthase 1 | Andean | ^21,23^ |
|  | *NOS3* | Nitric Oxide Synthase 3 | Andean | ^43^ |
|  | *NOTCH1* | Notch Receptor 1 | Andean | ^21,23^ |
|  | *NRP1* | Neuropilin 1 | Andean/ Himalayan | ^21,23^ |
|  | *NRP2* | Neuropilin 2 | Himalayan | ^21,23^ |
|  | *OTX1* | Orthodenticle Homeobox 1 | Himalayan | ^38^ |
|  | *OXR1* | Oxidation Resistance 1 | Himalayan | ^25^ |
|  | *P73* | Tumor Protein 73 | Himalayan | ^33^ |
|  | *PAFAHIB3* |  | Ethiopians | ^35^ |
|  | *PAPOLA* | Poly(A) Polymerase Alpha | Himalayan | ^31^ |
|  | *PDGF2* | Platelet Derived Growth Factor Subunit B | Himalayan | ^21^ |
|  | *PFKM* | Phosphofructokinase, Muscle | Andean | ^52^ |
|  | *PGF* | Placental Growth Factor | Himalayan | ^21^ |
|  | *PIK3CA* | Phosphatidylinositol-4,5-Bisphosphate 3-Kinase Catalytic Subunit Alpha | Andean | ^23^  ^40^ |
|  | *PIK3CB* | Phosphatidylinositol-4,5-Bisphosphate 3-Kinase Catalytic Subunit Beta | Himalayan | ^23^ |
|  | *PIK3CG* | Phosphatidylinositol-4,5-Bisphosphate 3-Kinase Catalytic Subunit Gamma | Andean | ^23^ |
|  | *PKLR* | Pyruvate kinase L/R | Himalayan | ^22,38^ |
|  | *POLR2A* | RNA Polymerase II Subunit A | Andean/ Himalayan | ^21,34^ |
|  | *PPA2* | Inorganic Pyrophosphatase 2 | Andean | ^37^ |
|  | *PPARA* | Peroxisome proliferator- activated receptor gamma coactivator 1-alpha | Himalayan | ^22,42^ |
|  | *PRKAA1* | Protein Kinase AMP-Activated Catalytic Subunit Alpha 1 | Andean | ^21,23^ |
|  | *PRKAA2* | Protein Kinase AMP-Activated Catalytic Subunit Alpha 2 | Andean | ^21,23^ |
|  | *PSMC3* | Proteasome 26S Subunit, ATPase 3 | Andean | ^21,23^ |
|  | *PTEN* | Phosphatase and Tensin Homolog | Himalayan | ^22^ |
|  | *PTGIS* | Prostaglandin I2 Synthase | Himalayan | ^47^ |
|  | *PYGM* | Glycogen Phosphorylase, Muscle Associated | Andean | ^37^ |
|  | *RBX1* | Ring-Box 1 | Himalayan | ^21^ |
|  | *RORA* | RAR Related Orphan Receptor A | Ethiopians | ^33^ |
|  | *RP11-384F7* | - | Himalayan | ^19^ |
|  | *RUNX1* | RUNX Family Transcription Factor 1 | Himalayan | ^26^ |
|  | *RYR1* | Ryanodine Receptor 1 | Himalayan | ^26^ |
|  | *SATB1* | SATB Homeobox 1 | Andean | ^23^  ^21^ |
|  | *SCUBE2* | Signal Peptide, CUB Domain and EGF Like Domain Containing 2 | Himalayan | ^51^ |
|  | *SFTPD* | Surfactant Protein D | Andean | ^46^ |
|  | *SGK3* | Serum/Glucocorticoid Regulated Kinase Family Member 3 | Andean | ^53^ |
|  | *SH2B1* | SH2B Adaptor Protein 1 | Andean | ^37^ |
|  | *SHISA6* | Shisa Family Member 6 | Andean | ^37^ |
|  | *SLC30A9* | Solute Carrier Family 30 Member 9 | Ethiopians  Himalayan | ^33,24^ |
|  | *SLC52A3* | Solute Carrier Family 52 Member 3 | Himalayan | ^19^ |
|  | *SMURF2* | SMAD Specific E3 Ubiquitin Protein Ligase 2 | Ethiopians | ^30^ |
|  | *SP100* | SP100 Nuclear Antigen | Andean | ^36^ |
|  | *SPRY2* | Sprouty RTK Signaling Antagonist 2 | Andean | ^21,23^ |
|  | *SPTA1* | Spectrin Alpha, Erythrocytic 1 | Himalayan | ^38^ |
|  | *STAT5B* | Signal Transducer and Activator of Transcription 5B | Himalayan | ^40^ |
|  | *TEX36* | Testis Expressed 36 | Himalayan | ^31^ |
|  | *TF* | Transferrin | Andean/ Himalayan | ^21,23^ |
|  | *TGFA* | Transforming Growth Factor Alpha | Andean | ^21,23,34,44^ |
|  | *TGFBR3* | Transforming Growth Factor Beta Receptor 3 | Himalayan | ^40^ |
|  | *THRB* | Thyroid Hormone Receptor Beta | Ethiopians | ^29^ |
|  | *TMEM247* | Transmembrane Protein 247 | Himalayan | ^25^ |
|  | *TMEM38B* | Transmembrane Protein 38B | Andean | ^36^ |
|  | *TNC* | Tenascin C | Andean/ Himalayan | ^22,21^ |
|  | *TNF* | Tumor Necrosis Factor | Andean/ Himalayan | ^21,23^ |
|  | *TP53* | Tumor Protein P53 | Andean | ^46^ |
|  | *TTC24* | Tetratricopeptide Repeat Domain 24 | Himalayan | ^25^ |
|  | *UBE2R2* | Ubiquitin Conjugating Enzyme E2 R2 | Ethiopians | ^35^ |
|  | *ULBP1* | UL16 Binding Protein 1 | Andean | ^37^ |
|  | *USP7* | Ubiquitin Specific Peptidase 7 | Andean | ^50^ |
|  | *VAV3* | Vav Guanine Nucleotide Exchange Factor 3 | Ethiopians | ^29^ |
|  | *VDR* | Vitamin D Receptor | Himalayan | ^47^ |
|  | *VEGFA* | Vascular endothelial growth factor A | Andean/ Himalayan | ^21,23,26^ |
|  | *VEGFB* | Vascular Endothelial Growth Factor B | Andean | ^32^ |
|  | *VEGFC* | Vascular Endothelial Growth Factor C | Himalayan | ^21^ |
|  | *VRK1* | VRK Serine/Threonine Kinase 1 | Himalayan | ^31^ |
|  | *ZNF532* | - | Himalayan | ^19^ |
|  | *ZNF644* | Zinc Finger Protein 644 | Himalayan | ^25^ |
| 164. | *RPIA* | Ribose 5-Phosphate Isomerase A | Tibetan | ^28^ |
| 165. | *NEK7* | NIMA Related Kinase 7 | Tibetan | ^28^ |
| 166. | *ADH7* | Alcohol Dehydrogenase 7 | Tibetan | ^28^ |
| 167. | *FGF10* | Fibroblast Growth Factor 10 | Tibetan | ^28^ |
| 168. | *HCAR2* | Hydroxycarboxylic Acid Receptor 2 | Tibetan | ^28^ |
| 169. | *HLA-DQB1* | Major Histocompatibility Complex, Class II, DQ Beta 1 | Tibetan | ^28^ |

**Table S3:** **List of proteins differentially expressed during human exposure to high altitude**. This combined list contains proteins (plasma and muscle proteins) for high altitude adaptation.

| **ID** | **Protein Name** | **Entrez Gene Name** | **Location** | **Type(s)** |
| --- | --- | --- | --- | --- |
| A1BG | A1BG | alpha-1-B glycoprotein | Extracellular Space | other |
| A2M | A2M | alpha-2-macroglobulin | Extracellular Space | transporter |
| P01023 | A2M | alpha-2-macroglobulin | Extracellular Space | transporter |
| ABCB9 | ABCB9 | ATP binding cassette subfamily B member 9 | Cytoplasm | transporter |
| ACTB | ACTB | actin beta | Cytoplasm | other |
| P63261 | ACTG1 | actin gamma 1 | Cytoplasm | other |
| ACTG2 | ACTG2 | actin gamma 2, smooth muscle | Cytoplasm | other |
| P63267 | ACTG2 | actin gamma 2, smooth muscle | Cytoplasm | other |
| ADAMTS20 | ADAMTS20 | ADAM metallopeptidase with thrombospondin type 1 motif 20 | Extracellular Space | peptidase |
| AHSG | AHSG | alpha 2-HS glycoprotein | Extracellular Space | other |
| ALB | ALB | albumin | Extracellular Space | transporter |
| ANG | ANG | angiogenin | Extracellular Space | enzyme |
| Q9BY76 | ANGPTL4 | angiopoietin like 4 | Extracellular Space | other |
| ANKRD20A4 | ANKRD20A4P (includes others) | ankyrin repeat domain 20 family member A3, pseudogene | Plasma Membrane | other |
| H3BPE8 | ANTKMT | adenine nucleotide translocase lysine methyltransferase | Cytoplasm | enzyme |
| P50995 | ANXA11 | annexin A11 | Nucleus | other |
| APCS | APCS | amyloid P component, serum | Extracellular Space | other |
| P02743 | APCS | amyloid P component, serum | Extracellular Space | other |
| H7C1U0 | APEH | acylaminoacyl-peptide hydrolase | Cytoplasm | peptidase |
| APOA1 | APOA1 | apolipoprotein A1 | Extracellular Space | transporter |
| P02647 | APOA1 | apolipoprotein A1 | Extracellular Space | transporter |
| APOA2 | APOA2 | apolipoprotein A2 | Extracellular Space | transporter |
| APOA4 | APOA4 | apolipoprotein A4 | Extracellular Space | transporter |
| APOA5 | APOA5 | apolipoprotein A5 | Extracellular Space | transporter |
| Q6Q788 | APOA5 | apolipoprotein A5 | Extracellular Space | transporter |
| APOC1 | APOC1 | apolipoprotein C1 | Extracellular Space | transporter |
| APOC2 | APOC2 | apolipoprotein C2 | Extracellular Space | transporter |
| APOE | APOE | apolipoprotein E | Extracellular Space | transporter |
| APOF | APOF | apolipoprotein F | Extracellular Space | transporter |
| APOH | APOH | apolipoprotein H | Extracellular Space | transporter |
| APOM | APOM | apolipoprotein M | Plasma Membrane | transporter |
| AQP6 | AQP6 | aquaporin 6 | Cytoplasm | transporter |
| B0YIW6 | ARCN1 | archain 1 | Cytoplasm | other |
| AZGP1 | AZGP1 | alpha-2-glycoprotein 1, zinc-binding | Extracellular Space | transporter |
| P20160 | AZU1 | azurocidin 1 | Cytoplasm | peptidase |
| S4R418 | BIN2 | bridging integrator 2 | Plasma Membrane | other |
| BLK | BLK | BLK proto-oncogene, Src family tyrosine kinase | Cytoplasm | kinase |
| Q8TDL5 | BPIFB1 | BPI fold containing family B member 1 | Extracellular Space | other |
| C1S | C1S | complement C1s | Extracellular Space | peptidase |
| P01024 | C3 | complement C3 | Extracellular Space | peptidase |
| P01024 | C3 | complement C3 | Extracellular Space | peptidase |
| P0C0L4 | C4A/C4B | complement C4A (Rodgers blood group) | Extracellular Space | other |
| C4BPA | C4BPA | complement component 4 binding protein alpha | Extracellular Space | other |
| C4BPB | C4BPB | complement component 4 binding protein beta | Extracellular Space | other |
| P01031 | C5 | complement C5 | Extracellular Space | cytokine |
| C8B | C8B | complement C8 beta chain | Extracellular Space | other |
| C8B | C8B | complement C8 beta chain | Extracellular Space | other |
| P02748 | C9 | complement C9 | Extracellular Space | other |
| F8WBR5 | CALM1 (includes others) | calmodulin 1 | Cytoplasm | other |
| P27797 | CALR | calreticulin | Cytoplasm | transcription regulator |
| O43852 | CALU | calumenin | Cytoplasm | other |
| A0A0C4DGH5 | CAND1 | cullin associated and neddylation dissociated 1 | Cytoplasm | transcription regulator |
| O95810 | CAVIN2 | caveolae associated protein 2 | Plasma Membrane | other |
| CCDC146 | CCDC146 | coiled-coil domain containing 146 | Cytoplasm | other |
| A0A087X089 | CCL14 | C-C motif chemokine ligand 14 | Extracellular Space | cytokine |
| P55774 | CCL18 | C-C motif chemokine ligand 18 | Extracellular Space | cytokine |
| CCT2 | CCT2 | chaperonin containing TCP1 subunit 2 | Cytoplasm | kinase |
| B4DUR8 | CCT3 | chaperonin containing TCP1 subunit 3 | Cytoplasm | other |
| F8WAM2 | CCT7 | chaperonin containing TCP1 subunit 7 | Cytoplasm | other |
| P60953 | CDC42 | cell division cycle 42 | Cytoplasm | enzyme |
| CFB | CFB | complement factor B | Extracellular Space | peptidase |
| CFD | CFD | complement factor D | Extracellular Space | peptidase |
| P10645 | CHGA | chromogranin A | Cytoplasm | other |
| O00299 | CLIC1 | chloride intracellular channel 1 | Nucleus | ion channel |
| F8WF69 | CLTA | clathrin light chain A | Plasma Membrane | other |
| A0A087WVQ6 | CLTC | clathrin heavy chain | Plasma Membrane | other |
| B4DDF4 | CNN2 | calponin 2 | Cytoplasm | other |
| H7C3T0 | COPS6 | COP9 signalosome subunit 6 | Nucleus | other |
| P31146 | CORO1A | coronin 1A | Cytoplasm | other |
| P22792 | CPN2 | carboxypeptidase N subunit 2 | Extracellular Space | peptidase |
| P24387 | CRHBP | corticotropin releasing hormone binding protein | Extracellular Space | other |
| J3KST8 | CRLF3 | cytokine receptor like factor 3 | Cytoplasm | transcription regulator |
| CST3 | CST3 | cystatin C | Extracellular Space | other |
| P08311 | CTSG | cathepsin G | Cytoplasm | peptidase |
| H0Y5J4 | DBNL | drebrin like | Cytoplasm | other |
| H0YII4 | DDI2 | DNA damage inducible 1 homolog 2 | Plasma Membrane | transporter |
| DSC1 | DSC1 | desmocollin 1 | Plasma Membrane | other |
| P26641 | EEF1G | eukaryotic translation elongation factor 1 gamma | Cytoplasm | translation regulator |
| EFHD1 | EFHD1 | EF-hand domain family member D1 | Cytoplasm | other |
| C9JDQ8 | EHD1 | EH domain containing 1 | Cytoplasm | other |
| J3KTN0 | EIF4A1 | eukaryotic translation initiation factor 4A1 | Cytoplasm | translation regulator |
| F8WCJ1 | EIF5A2 | eukaryotic translation initiation factor 5A2 | Cytoplasm | translation regulator |
| ERCC5 | ERCC5 | ERCC excision repair 5, endonuclease | Nucleus | enzyme |
| F10 | F10 | coagulation factor X | Extracellular Space | peptidase |
| P03951 | F11 | coagulation factor XI | Extracellular Space | peptidase |
| F13B | F13B | coagulation factor XIII B chain | Extracellular Space | enzyme |
| F2 | F2 | coagulation factor II, prothrombin | Extracellular Space | peptidase |
| F7 | F7 | coagulation factor VII | Plasma Membrane | peptidase |
| F9 | F9 | coagulation factor IX | Extracellular Space | peptidase |
| Q9Y3I1 | FBXO7 | F-box protein 7 | Cytoplasm | enzyme |
| FCN2 | FCN2 | ficolin 2 | Extracellular Space | other |
| Q15485 | FCN2 | ficolin 2 | Extracellular Space | other |
| FCN3 | FCN3 | ficolin 3 | Extracellular Space | other |
| FGA | FGA | fibrinogen alpha chain | Extracellular Space | other |
| FIGNL1 | FIGNL1 | fidgetin like 1 | Nucleus | enzyme |
| FRK | FRK | fyn related Src family tyrosine kinase | Nucleus | kinase |
| FRMD7 | FRMD7 | FERM domain containing 7 | Plasma Membrane | other |
| E9PD92 | G6PD | glucose-6-phosphate dehydrogenase | Cytoplasm | enzyme |
| GAPDH | GAPDH | glyceraldehyde-3-phosphate dehydrogenase | Cytoplasm | enzyme |
| P04406 | GAPDH | glyceraldehyde-3-phosphate dehydrogenase | Cytoplasm | enzyme |
| GAPDH | GAPDH | glyceraldehyde-3-phosphate dehydrogenase | Cytoplasm | enzyme |
| GC | GC | GC vitamin D binding protein | Extracellular Space | transporter |
| D6RF35 | GC | GC vitamin D binding protein | Extracellular Space | transporter |
| P02774 | GC | GC vitamin D binding protein | Extracellular Space | transporter |
| P02774 | GC | GC vitamin D binding protein | Extracellular Space | transporter |
| M0QYG8 | GMFG | glia maturation factor gamma | Cytoplasm | growth factor |
| GPLD1 | GPLD1 | glycosylphosphatidylinositol specific phospholipase D1 | Cytoplasm | enzyme |
| GPN1 | GPN1 | GPN-loop GTPase 1 | Nucleus | transcription regulator |
| GPX3 | GPX3 | glutathione peroxidase 3 | Extracellular Space | enzyme |
| A0A087X1J7 | GPX3 | glutathione peroxidase 3 | Extracellular Space | enzyme |
| GSN | GSN | gelsolin | Extracellular Space | other |
| P78417 | GSTO1 | glutathione S-transferase omega 1 | Cytoplasm | enzyme |
| GSTP1 | GSTP1 | glutathione S-transferase pi 1 | Cytoplasm | enzyme |
| P16403 | H1-2 | H1.2 linker histone, cluster member | Nucleus | other |
| P16401 | H1-5 | H1.5 linker histone, cluster member | Nucleus | other |
| HBB | HBB | hemoglobin subunit beta | Cytoplasm | transporter |
| P68871 | HBB | hemoglobin subunit beta | Cytoplasm | transporter |
| HGFAC | HGFAC | HGF activator | Extracellular Space | peptidase |
| P05534 | HLA-A | major histocompatibility complex, class I, A | Plasma Membrane | other |
| HP | HP | haptoglobin | Extracellular Space | peptidase |
| P00738 | HP | haptoglobin | Extracellular Space | peptidase |
| Q9Y251 | HPSE | heparanase | Plasma Membrane | enzyme |
| HPX | HPX | hemopexin | Extracellular Space | transporter |
| P02790 | HPX | hemopexin | Extracellular Space | transporter |
| P04196 | HRG | histidine rich glycoprotein | Extracellular Space | other |
| P07900 | HSP90AA1 | heat shock protein 90 alpha family class A member 1 | Cytoplasm | enzyme |
| P08238 | HSP90AB1 | heat shock protein 90 alpha family class B member 1 | Cytoplasm | enzyme |
| P04792 | HSPB1 | heat shock protein family B (small) member 1 | Cytoplasm | other |
| IGFBP1 | IGFBP1 | insulin like growth factor binding protein 1 | Extracellular Space | other |
| C9JXF9 | IGFBP1 | insulin like growth factor binding protein 1 | Extracellular Space | other |
| P18065 | IGFBP2 | insulin like growth factor binding protein 2 | Extracellular Space | other |
| A0A0J9YXX1 | IGHV5-10-1 | immunoglobulin heavy variable 5-10-1 | Other | other |
| INHBE | INHBE | inhibin subunit beta E | Extracellular Space | growth factor |
| P46940 | IQGAP1 | IQ motif containing GTPase activating protein 1 | Cytoplasm | other |
| P08514 | ITGA2B | integrin subunit alpha 2b | Plasma Membrane | transmembrane receptor |
| ITIH1 | ITIH1 | inter-alpha-trypsin inhibitor heavy chain 1 | Extracellular Space | other |
| ITIH2 | ITIH2 | inter-alpha-trypsin inhibitor heavy chain 2 | Extracellular Space | other |
| ITM2B | ITM2B | integral membrane protein 2B | Plasma Membrane | other |
| B7WPD9 | KIF26B | kinesin family member 26B | Other | other |
| Q14974 | KPNB1 | karyopherin subunit beta 1 | Nucleus | other |
| KRT10 | KRT10 | keratin 10 | Cytoplasm | other |
| KRT14 | KRT14 | keratin 14 | Cytoplasm | other |
| KRT6B | KRT6B | keratin 6B | Cytoplasm | other |
| KRT71 | KRT71 | keratin 71 | Cytoplasm | other |
| KRT76 | KRT76 | keratin 76 | Cytoplasm | other |
| KRTDAP | KRTDAP | keratinocyte differentiation associated protein | Extracellular Space | other |
| Q14847 | LASP1 | LIM and SH3 protein 1 | Cytoplasm | transporter |
| LCAT | LCAT | lecithin-cholesterol acyltransferase | Extracellular Space | enzyme |
| P13796 | LCP1 | lymphocyte cytosolic protein 1 | Cytoplasm | other |
| LGALS3 | LGALS3 | galectin 3 | Extracellular Space | other |
| E7EUK6 | LIPC | lipase C, hepatic type | Extracellular Space | enzyme |
| LRG1 | LRG1 | leucine rich alpha-2-glycoprotein 1 | Extracellular Space | other |
| P09960 | LTA4H | leukotriene A4 hydrolase | Cytoplasm | enzyme |
| E7EQB2 | LTF | lactotransferrin | Extracellular Space | peptidase |
| P33908 | MAN1A1 | mannosidase alpha class 1A member 1 | Cytoplasm | enzyme |
| MB | MB | myoglobin | Cytoplasm | transporter |
| Q96HR3 | MED30 | mediator complex subunit 30 | Nucleus | transcription regulator |
| E7EPG1 | MMRN1 | multimerin 1 | Extracellular Space | other |
| P05164 | MPO | myeloperoxidase | Cytoplasm | enzyme |
| Q7Z406 | MYH14 | myosin heavy chain 14 | Extracellular Space | enzyme |
| P35579 | MYH9 | myosin heavy chain 9 | Cytoplasm | enzyme |
| J3QRS3 | MYL12A | myosin light chain 12A | Cytoplasm | other |
| MYOM1 | MYOM1 | myomesin 1 | Cytoplasm | other |
| H0YHC3 | NAP1L1 | nucleosome assembly protein 1 like 1 | Nucleus | other |
| J3KPD9 | NME1-NME2 | NME1-NME2 readthrough | Cytoplasm | other |
| F2Z2K0 | NSFL1C | NSFL1 cofactor | Cytoplasm | other |
| Q8WWZ8 | OIT3 | oncoprotein induced transcript 3 | Nucleus | other |
| ORM2 | ORM2 | orosomucoid 2 | Extracellular Space | other |
| Q92882 | OSTF1 | osteoclast stimulating factor 1 | Nucleus | transcription regulator |
| P13667 | PDIA4 | protein disulfide isomerase family A member 4 | Cytoplasm | enzyme |
| Q15084 | PDIA6 | protein disulfide isomerase family A member 6 | Cytoplasm | enzyme |
| O00151 | PDLIM1 | PDZ and LIM domain 1 | Cytoplasm | transcription regulator |
| Q9NR12 | PDLIM7 | PDZ and LIM domain 7 | Cytoplasm | other |
| PF4 | PF4 | platelet factor 4 | Extracellular Space | cytokine |
| P02776 | PF4 | platelet factor 4 | Extracellular Space | cytokine |
| P14618 | PKM | pyruvate kinase M1/2 | Cytoplasm | kinase |
| PLEK | PLEK | pleckstrin | Cytoplasm | other |
| PLTP | PLTP | phospholipid transfer protein | Extracellular Space | enzyme |
| PON1 | PON1 | paraoxonase 1 | Extracellular Space | phosphatase |
| PON3 | PON3 | paraoxonase 3 | Extracellular Space | enzyme |
| POTEC | POTEC | POTE ankyrin domain family member C | Plasma Membrane | other |
| PPBP | PPBP | pro-platelet basic protein | Extracellular Space | cytokine |
| P02775 | PPBP | pro-platelet basic protein | Extracellular Space | cytokine |
| P62937 | PPIA | peptidylprolyl isomerase A | Cytoplasm | enzyme |
| PPL | PPL | periplakin | Cytoplasm | other |
| B3KQV6 | PPP2R1A | protein phosphatase 2 scaffold subunit Aalpha | Cytoplasm | phosphatase |
| A0A0A0MSI0 | PRDX1 | peroxiredoxin 1 | Cytoplasm | enzyme |
| P32119 | PRDX2 | peroxiredoxin 2 | Cytoplasm | enzyme |
| P30044 | PRDX5 | peroxiredoxin 5 | Cytoplasm | enzyme |
| P30041 | PRDX6 | peroxiredoxin 6 | Cytoplasm | enzyme |
| K7ELL7 | PRKCSH | protein kinase C substrate 80K-H | Cytoplasm | enzyme |
| PROC | PROC | protein C, inactivator of coagulation factors Va and VIIIa | Extracellular Space | peptidase |
| PROS1 | PROS1 | protein S | Extracellular Space | other |
| PROZ | PROZ | protein Z, vitamin K dependent plasma glycoprotein | Extracellular Space | peptidase |
| P62195 | PSMC5 | proteasome 26S subunit, ATPase 5 | Nucleus | transcription regulator |
| H7C378 | PSMD1 | proteasome 26S subunit, non-ATPase 1 | Cytoplasm | other |
| H0Y6Z7 | PTPRF | protein tyrosine phosphatase receptor type F | Plasma Membrane | phosphatase |
| Q00577 | PURA | purine rich element binding protein A | Nucleus | transcription regulator |
| E9PLD0 | RAB1B | RAB1B, member RAS oncogene family | Cytoplasm | other |
| B1AH77 | RAC2 | Rac family small GTPase 2 | Cytoplasm | enzyme |
| RBP4 | RBP4 | retinol binding protein 4 | Extracellular Space | other |
| P02753 | RBP4 | retinol binding protein 4 | Extracellular Space | other |
| Q15293 | RCN1 | reticulocalbin 1 | Cytoplasm | other |
| Q9H4X1 | RGCC | regulator of cell cycle | Cytoplasm | other |
| RNASE4 | RNASE4 | ribonuclease A family member 4 | Extracellular Space | enzyme |
| P13489 | RNH1 | ribonuclease/angiogenin inhibitor 1 | Cytoplasm | other |
| H0YDD8 | RPLP2 | ribosomal protein lateral stalk subunit P2 | Cytoplasm | other |
| F6U211 | RPS10 | ribosomal protein S10 | Cytoplasm | other |
| P62701 | RPS4X | ribosomal protein S4 X-linked | Cytoplasm | other |
| Q15404 | RSU1 | Ras suppressor protein 1 | Cytoplasm | other |
| P31151 | S100A7 | S100 calcium binding protein A7 | Cytoplasm | other |
| S100A8 | S100A8 | S100 calcium binding protein A8 | Cytoplasm | other |
| P05109 | S100A8 | S100 calcium binding protein A8 | Cytoplasm | other |
| P06702 | S100A9 | S100 calcium binding protein A9 | Cytoplasm | other |
| A0A182DWH7 | SELENOP | selenoprotein P | Extracellular Space | other |
| P01009 | SERPINA1 | serpin family A member 1 | Extracellular Space | other |
| SERPINA6 | SERPINA6 | serpin family A member 6 | Extracellular Space | other |
| SERPIND1 | SERPIND1 | serpin family D member 1 | Extracellular Space | other |
| P05546 | SERPIND1 | serpin family D member 1 | Extracellular Space | other |
| SERPINE1 | SERPINE1 | serpin family E member 1 | Extracellular Space | other |
| SERPINF1 | SERPINF1 | serpin family F member 1 | Extracellular Space | other |
| P08697 | SERPINF2 | serpin family F member 2 | Extracellular Space | other |
| E5RJR5 | SKP1 | S-phase kinase associated protein 1 | Nucleus | transcription regulator |
| P09486 | SPARC | secreted protein acidic and cysteine rich | Extracellular Space | other |
| SPARCL1 | SPARCL1 | SPARC like 1 | Extracellular Space | other |
| SPP2 | SPP2 | secreted phosphoprotein 2 | Extracellular Space | other |
| H7C3I1 | ST13 | ST13 Hsp70 interacting protein | Cytoplasm | other |
| TAF1L | TAF1L | TATA-box binding protein associated factor 1 like | Nucleus | transcription regulator |
| X6RJP6 | TAGLN2 | transgelin 2 | Cytoplasm | other |
| TF | TF | transferrin | Extracellular Space | transporter |
| P02787 | TF | transferrin | Extracellular Space | transporter |
| THAP4 | THAP4 | THAP domain containing 4 | Nucleus | transcription regulator |
| THBS1 | THBS1 | thrombospondin 1 | Extracellular Space | other |
| H0YKU1 | TMOD3 | tropomodulin 3 | Cytoplasm | other |
| Q6ZN40 | TPM1 | tropomyosin 1 | Cytoplasm | other |
| P02766 | TTR | transthyretin | Extracellular Space | transporter |
| P68366 | TUBA4A | tubulin alpha 4a | Cytoplasm | other |
| Q5JP53 | TUBB | tubulin beta class I | Cytoplasm | other |
| P68371 | TUBB4B | tubulin beta 4B class IVb | Cytoplasm | other |
| O43396 | TXNL1 | thioredoxin like 1 | Cytoplasm | enzyme |
| P45974 | USP5 | ubiquitin specific peptidase 5 | Cytoplasm | peptidase |
| P50552 | VASP | vasodilator stimulated phosphoprotein | Plasma Membrane | other |
| B0YJC4 | VIM | vimentin | Cytoplasm | other |
| P62258 | YWHAE | tyrosine 3-monooxygenase/tryptophan 5-monooxygenase activation protein epsilon | Cytoplasm | other |
| Q04917 | YWHAH | tyrosine 3-monooxygenase/tryptophan 5-monooxygenase activation protein eta | Cytoplasm | transcription regulator |
| P27348 | YWHAQ | tyrosine 3-monooxygenase/tryptophan 5-monooxygenase activation protein theta | Cytoplasm | other |
| YWHAZ | YWHAZ | tyrosine 3-monooxygenase/tryptophan 5-monooxygenase activation protein zeta | Cytoplasm | enzyme |
| P63104 | YWHAZ | tyrosine 3-monooxygenase/tryptophan 5-monooxygenase activation protein zeta | Cytoplasm | enzyme |
| ZNF573 | ZNF573 | zinc finger protein 573 | Nucleus | other |
| H0Y2Y8 | ZYX | zyxin | Plasma Membrane | other |

**References**

(1) Viganò, A.; Ripamonti, M.; De Palma, S.; Capitanio, D.; Vasso, M.; Wait, R.; Lundby, C.; Cerretelli, P.; Gelfi, C. Proteins modulation in human skeletal muscle in the early phase of adaptation to hypobaric hypoxia. *Proteomics* **2008**, *8* (22), 4668-4679.

(2) Levett, D. Z.; Viganò, A.; Capitanio, D.; Vasso, M.; De Palma, S.; Moriggi, M.; Martin, D. S.; Murray, A. J.; Cerretelli, P.; Grocott, M. P. Changes in muscle proteomics in the course of the Caudwell Research Expedition to Mt. Everest. *Proteomics* **2015**, *15* (1), 160-171.

(3) Jain, S.; Paul, S.; Meena, R. N.; Gangwar, A.; Panjwani, U.; Ahmad, Y.; Bhargava, K. Saliva panel of protein candidates: A comprehensive study for assessing high altitude acclimatization. *Nitric Oxide* **2020**, *95*, 1-11.

(4) Gangwar, A.; Sharma, M.; Singh, K.; Patyal, A.; Bhaumik, G.; Bhargava, K.; Sethy, N. K. Intermittent normobaric hypoxia facilitates high altitude acclimatization by curtailing hypoxia-induced inflammation and dyslipidemia. *Pflügers Archiv-European Journal of Physiology* **2019**, *471* (7), 949-959.

(5) Jain, S.; Ahmad, Y.; Bhargava, K. Salivary proteome patterns of individuals exposed to High Altitude. *Archives of oral biology* **2018**, *96*, 104-112.

(6) Hinkelbein, J.; Jansen, S.; Iovino, I.; Kruse, S.; Meyer, M.; Cirillo, F.; Drinhaus, H.; Hohn, A.; Klein, C.; Robertis, E. D. Thirty minutes of hypobaric hypoxia provokes alterations of immune response, haemostasis, and metabolism proteins in human serum. *International journal of molecular sciences* **2017**, *18* (9), 1882.

(7) Julian, C. G.; Subudhi, A. W.; Hill, R. C.; Wilson, M. J.; Dimmen, A. C.; Hansen, K. C.; Roach, R. C. Exploratory proteomic analysis of hypobaric hypoxia and acute mountain sickness in humans. *J Appl Physiol (1985)* **2014**, *116* (7), 937-944. DOI: 10.1152/japplphysiol.00362.2013.

(8) Ahmad, Y.; Sharma, N. K.; Garg, I.; Ahmad, M. F.; Sharma, M.; Bhargava, K. An insight into the changes in human plasma proteome on adaptation to hypobaric hypoxia. *PloS one* **2013**, *8* (7), e67548.

(9) Yang, Y.; Ma, L.; Guan, W.; Wang, Y.; DU, Y.; Ga, Q.; Ge, R. L. Differential plasma proteome analysis in patients with high-altitude pulmonary edema at the acute and recovery phases. *Exp Ther Med* **2014**, *7* (5), 1160-1166. DOI: 10.3892/etm.2014.1548.

(10) Zhang, Y. Y.; Duan, R. F.; Cui, W. Y.; Pan, Z. Y.; Liu, W.; Long, C. L.; Wang, Y. H.; Wang, H. Proteomic identification of human serum biomarkers associated with high altitude pulmonary edema. *Zhongguo Ying Yong Sheng Li Xue Za Zhi* **2013**, *29* (6), 501-507.

(11) Levett, D. Z.; Radford, E. J.; Menassa, D. A.; Graber, E. F.; Morash, A. J.; Hoppeler, H.; Clarke, K.; Martin, D. S.; Ferguson‐Smith, A. C.; Montgomery, H. E. Acclimatization of skeletal muscle mitochondria to high‐altitude hypoxia during an ascent of Everest. *The FASEB journal* **2012**, *26* (4), 1431-1441.

(12) Du, X.; Zhang, R.; Ye, S.; Liu, F.; Jiang, P.; Yu, X.; Xu, J.; Ma, L.; Cao, H.; Shen, Y. Alterations of human plasma proteome profile on adaptation to high-altitude hypobaric hypoxia. *Journal of proteome research* **2019**, *18* (5), 2021-2031.

(13) Chicco, A. J.; Le, C. H.; Gnaiger, E.; Dreyer, H. C.; Muyskens, J. B.; D'Alessandro, A.; Nemkov, T.; Hocker, A. D.; Prenni, J. E.; Wolfe, L. M. Adaptive remodeling of skeletal muscle energy metabolism in high-altitude hypoxia: lessons from AltitudeOmics. *Journal of Biological Chemistry* **2018**, *293* (18), 6659-6671.

(14) Horscroft, J. A.; Kotwica, A. O.; Laner, V.; West, J. A.; Hennis, P. J.; Levett, D. Z.; Howard, D. J.; Fernandez, B. O.; Burgess, S. L.; Ament, Z. Metabolic basis to Sherpa altitude adaptation. *Proceedings of the National Academy of Sciences* **2017**, *114* (24), 6382-6387.

(15) Wang, Z.; Liu, F.; Ye, S.; Jiang, P.; Yu, X.; Xu, J.; Du, X.; Ma, L.; Cao, H.; Yuan, C. Plasma proteome profiling of high-altitude polycythemia using TMT-based quantitative proteomics approach. *Journal of proteomics* **2019**, *194*, 60-69.

(16) Gelfi, C.; De Palma, S.; Ripamonti, M.; Wait, R.; Eberini, I.; Bajracharya, A.; Marconi, C.; Schneider, A.; Hoppeler, H.; Cerretelli, P. New aspects of altitude adaptation in Tibetans: a proteomic approach. *The FASEB journal* **2004**, *18* (3), 612-614.

(17) Yang, J.; Li, W.; Liu, S.; Yuan, D.; Guo, Y.; Jia, C.; Song, T.; Huang, C. Identification of novel serum peptide biomarkers for high-altitude adaptation: a comparative approach. *Scientific reports* **2016**, *6* (1), 1-9.

(18) Mainini, V.; Gianazza, E.; Chinello, C.; Bilo, G.; Revera, M.; Giuliano, A.; Caldara, G.; Lombardi, C.; Piperno, A.; Magni, F. Modulation of urinary peptidome in humans exposed to high altitude hypoxia. *Molecular BioSystems* **2012**, *8* (4), 959-966.

(19) Arciero, E.; Kraaijenbrink, T.; Haber, M.; Mezzavilla, M.; Ayub, Q.; Wang, W.; Pingcuo, Z.; Yang, H.; Wang, J.; Jobling, M. A. Demographic history and genetic adaptation in the Himalayan region inferred from genome-wide SNP genotypes of 49 populations. *Molecular biology and evolution* **2018**, *35* (8), 1916-1933.

(20) Droma, Y.; Hanaoka, M.; Basnyat, B.; Arjyal, A.; Neupane, P.; Pandit, A.; Sharma, D.; Ito, M.; Miwa, N.; Katsuyama, Y. Adaptation to high altitude in Sherpas: association with the insertion/deletion polymorphism in the angiotensin-converting enzyme gene. *Wilderness & environmental medicine* **2008**, *19* (1), 22-29.

(21) Bigham, A.; Bauchet, M.; Pinto, D.; Mao, X.; Akey, J. M.; Mei, R.; Scherer, S. W.; Julian, C. G.; Wilson, M. J.; Herráez, D. L. Identifying signatures of natural selection in Tibetan and Andean populations using dense genome scan data. *PLoS Genet* **2010**, *6* (9), e1001116.

(22) Simonson, T. S.; Yang, Y.; Huff, C. D.; Yun, H.; Qin, G.; Witherspoon, D. J.; Bai, Z.; Lorenzo, F. R.; Xing, J.; Jorde, L. B. Genetic evidence for high-altitude adaptation in Tibet. *Science* **2010**, *329* (5987), 72-75.

(23) Bigham, A. W.; Mao, X.; Mei, R.; Brutsaert, T.; Wilson, M. J.; Julian, C. G.; Parra, E. J.; Akey, J. M.; Moore, L. G.; Shriver, M. D. Identifying positive selection candidate loci for high-altitude adaptation in Andean populations. *Human genomics* **2009**, *4* (2), 79.

(24) Alkorta-Aranburu, G.; Beall, C. M.; Witonsky, D. B.; Gebremedhin, A.; Pritchard, J. K.; Di Rienzo, A. The genetic architecture of adaptations to high altitude in Ethiopia. *PLoS Genet* **2012**, *8* (12), e1003110.

(25) Zhang, C.; Lu, Y.; Feng, Q.; Wang, X.; Lou, H.; Liu, J.; Ning, Z.; Yuan, K.; Wang, Y.; Zhou, Y. Differentiated demographic histories and local adaptations between Sherpas and Tibetans. *Genome biology* **2017**, *18* (1), 1-18.

(26) Wang, B.; Zhang, Y.-B.; Zhang, F.; Lin, H.; Wang, X.; Wan, N.; Ye, Z.; Weng, H.; Zhang, L.; Li, X. On the origin of Tibetans and their genetic basis in adapting high-altitude environments. *PloS one* **2011**, *6* (2), e17002.

(27) Beall, C. M.; Cavalleri, G. L.; Deng, L.; Elston, R. C.; Gao, Y.; Knight, J.; Li, C.; Li, J. C.; Liang, Y.; McCormack, M. Natural selection on EPAS1 (HIF2α) associated with low hemoglobin concentration in Tibetan highlanders. *Proceedings of the National Academy of Sciences* **2010**, *107* (25), 11459-11464.

(28) Yang, J.; Jin, Z.-B.; Chen, J.; Huang, X.-F.; Li, X.-M.; Liang, Y.-B.; Mao, J.-Y.; Chen, X.; Zheng, Z.; Bakshi, A. Genetic signatures of high-altitude adaptation in Tibetans. *Proceedings of the National Academy of Sciences* **2017**, *114* (16), 4189-4194.

(29) Scheinfeldt, L. B.; Soi, S.; Thompson, S.; Ranciaro, A.; Woldemeskel, D.; Beggs, W.; Lambert, C.; Jarvis, J. P.; Abate, D.; Belay, G. Genetic adaptation to high altitude in the Ethiopian highlands. *Genome biology* **2012**, *13* (1), 1-9.

(30) Huerta-Sánchez, E.; DeGiorgio, M.; Pagani, L.; Tarekegn, A.; Ekong, R.; Antao, T.; Cardona, A.; Montgomery, H. E.; Cavalleri, G. L.; Robbins, P. A. Genetic signatures reveal high-altitude adaptation in a set of Ethiopian populations. *Molecular biology and evolution* **2013**, *30* (8), 1877-1888.

(31) Jeong, C.; Witonsky, D. B.; Basnyat, B.; Neupane, M.; Beall, C. M.; Childs, G.; Craig, S. R.; Novembre, J.; Di Rienzo, A. Detecting past and ongoing natural selection among ethnically Tibetan women at high altitude in Nepal. *PLoS genetics* **2018**, *14* (9), e1007650.

(32) Eichstaedt, C. A.; Antão, T.; Pagani, L.; Cardona, A.; Kivisild, T.; Mormina, M. The Andean adaptive toolkit to counteract high altitude maladaptation: genome-wide and phenotypic analysis of the Collas. *PloS one* **2014**, *9* (3), e93314.

(33) Gnecchi-Ruscone, G. A.; Abondio, P.; De Fanti, S.; Sarno, S.; Sherpa, M. G.; Sherpa, P. T.; Marinelli, G.; Natali, L.; Di Marcello, M.; Peluzzi, D. Evidence of polygenic adaptation to high altitude from Tibetan and Sherpa genomes. *Genome biology and evolution* **2018**, *10* (11), 2919-2930.

(34) Bigham, A. W.; Lee, F. S. Human high-altitude adaptation: forward genetics meets the HIF pathway. *Genes & development* **2014**, *28* (20), 2189-2204.

(35) Udpa, N.; Ronen, R.; Zhou, D.; Liang, J.; Stobdan, T.; Appenzeller, O.; Yin, Y.; Du, Y.; Guo, L.; Cao, R. Whole genome sequencing of Ethiopian highlanders reveals conserved hypoxia tolerance genes. *Genome biology* **2014**, *15* (2), 1-14.

(36) Jacovas, V. C.; Couto-Silva, C. M.; Nunes, K.; Lemes, R. B.; de Oliveira, M. Z.; Salzano, F. M.; Bortolini, M. C.; Hünemeier, T. Selection scan reveals three new loci related to high altitude adaptation in Native Andeans. *Scientific reports* **2018**, *8* (1), 1-8.

(37) Crawford, J. E.; Amaru, R.; Song, J.; Julian, C. G.; Racimo, F.; Cheng, J. Y.; Guo, X.; Yao, J.; Ambale-Venkatesh, B.; Lima, J. A. Natural selection on genes related to cardiovascular health in high-altitude adapted Andeans. *The American Journal of Human Genetics* **2017**, *101* (5), 752-767.

(38) Yi, X.; Liang, Y.; Huerta-Sanchez, E.; Jin, X.; Cuo, Z. X. P.; Pool, J. E.; Xu, X.; Jiang, H.; Vinckenbosch, N.; Korneliussen, T. S. Sequencing of 50 human exomes reveals adaptation to high altitude. *Science* **2010**, *329* (5987), 75-78.

(39) Lindo, J.; Haas, R.; Hofman, C.; Apata, M.; Moraga, M.; Verdugo, R. A.; Watson, J. T.; Llave, C. V.; Witonsky, D.; Beall, C. The genetic prehistory of the Andean highlands 7000 years BP though European contact. *Science advances* **2018**, *4* (11), eaau4921.

(40) Peng, Y.; Yang, Z.; Zhang, H.; Cui, C.; Qi, X.; Luo, X.; Tao, X.; Wu, T.; Chen, H.; Shi, H. Genetic variations in Tibetan populations and high-altitude adaptation at the Himalayas. *Molecular biology and evolution* **2011**, *28* (2), 1075-1081.

(41) Xu, S.; Li, S.; Yang, Y.; Tan, J.; Lou, H.; Jin, W.; Yang, L.; Pan, X.; Wang, J.; Shen, Y. A genome-wide search for signals of high-altitude adaptation in Tibetans. *Molecular biology and evolution* **2011**, *28* (2), 1003-1011.

(42) Simonson, T.; Huff, C.; Witherspoon, D.; Prchal, J.; Jorde, L. Adaptive genetic changes related to haemoglobin concentration in native high‐altitude Tibetans. *Experimental physiology* **2015**, *100* (11), 1263-1268.

(43) Fehren-Schmitz, L.; Georges, L. Ancient DNA reveals selection acting on genes associated with hypoxia response in pre-Columbian Peruvian Highlanders in the last 8500 years. *Scientific reports* **2016**, *6* (1), 1-11.

(44) Bigham, A. W.; Wilson, M. J.; Julian, C. G.; Kiyamu, M.; Vargas, E.; Leon‐Velarde, F.; Rivera‐Chira, M.; Rodriquez, C.; Browne, V. A.; Parra, E. Andean and Tibetan patterns of adaptation to high altitude. *American Journal of Human Biology* **2013**, *25* (2), 190-197.

(45) Moore, L. G.; Shriver, M.; Bemis, L.; Hickler, B.; Wilson, M.; Brutsaert, T.; Parra, E.; Vargas, E. Maternal adaptation to high-altitude pregnancy: an experiment of nature—a review. *Placenta* **2004**, *25*, S60-S71.

(46) Valverde, G.; Zhou, H.; Lippold, S.; de Filippo, C.; Tang, K.; Herráez, D. L.; Li, J.; Stoneking, M. A novel candidate region for genetic adaptation to high altitude in Andean populations. *PLoS One* **2015**, *10* (5), e0125444.

(47) Hu, H.; Petousi, N.; Glusman, G.; Yu, Y.; Bohlender, R.; Tashi, T.; Downie, J. M.; Roach, J. C.; Cole, A. M.; Lorenzo, F. R. Evolutionary history of Tibetans inferred from whole-genome sequencing. *PLoS genetics* **2017**, *13* (4), e1006675.

(48) Guo, Y.-B.; He, Y.-X.; Cui, C.-Y. GCH1 plays a role in the high-altitude adaptation of Tibetans. *Zoological Research* **2017**, *38* (3), 155.

(49) Jeong, C.; Alkorta-Aranburu, G.; Basnyat, B.; Neupane, M.; Witonsky, D. B.; Pritchard, J. K.; Beall, C. M.; Di Rienzo, A. Admixture facilitates genetic adaptations to high altitude in Tibet. *Nature communications* **2014**, *5* (1), 1-7.

(50) Jacovas, V. C.; Rovaris, D. L.; Peréz, O.; de Azevedo, S.; Macedo, G. S.; Sandoval, J. R.; Salazar-Granara, A.; Villena, M.; Dugoujon, J.-M.; Bisso-Machado, R. Genetic variations in the TP53 pathway in Native Americans strongly suggest adaptation to the high altitudes of the Andes. *PLoS One* **2015**, *10* (9), e0137823.

(51) Ouzhuluobu; He, Y.; Lou, H.; Cui, C.; Deng, L.; Gao, Y.; Zheng, W.; Guo, Y.; Wang, X.; Ning, Z. De novo assembly of a Tibetan genome and identification of novel structural variants associated with high-altitude adaptation. *National Science Review* **2020**, *7* (2), 391-402.

(52) Zhou, D.; Udpa, N.; Ronen, R.; Stobdan, T.; Liang, J.; Appenzeller, O.; Zhao, H. W.; Yin, Y.; Du, Y.; Guo, L. Whole-genome sequencing uncovers the genetic basis of chronic mountain sickness in Andean highlanders. *The American Journal of Human Genetics* **2013**, *93* (3), 452-462.

(53) Stobdan, T.; Akbari, A.; Azad, P.; Zhou, D.; Poulsen, O.; Appenzeller, O.; Gonzales, G. F.; Telenti, A.; Wong, E. H.; Saini, S. New insights into the genetic basis of Monge’s disease and adaptation to high-altitude. *Molecular biology and evolution* **2017**, *34* (12), 3154-3168.
